# Structural insights into GPCR signaling activated by peptide ligands: from molecular mechanism to therapeutic application

**DOI:** 10.1038/s12276-025-01497-y

**Published:** 2025-07-08

**Authors:** Jinuk Kim, Jeesoo Kim, Chulwon Choi, Jungnam Bae, Hee-Jung Choi

**Affiliations:** 1 Department of Biological Sciences, Seoul National University, Seoul, Republic of Korea; 2 Present Address: Division of Biological Science and Technology, Yonsei University, Wonju, Republic of Korea

**Keywords:** Biochemistry, Drug discovery, Biotechnology

## Abstract

Recent advances in structural biology have profoundly enhanced our understanding of G protein-coupled receptors (GPCRs), providing detailed molecular insights into their activation and ligand recognition. Here, in this Review, we explore the molecular mechanisms of class A and class B GPCRs bound to peptide agonists and their implications for drug development. We examine representative GPCRs, such as the angiotensin II type 1 receptor, chemokine receptor 5, μ-opioid receptor, parathyroid hormone 1 receptor and glucagon-like peptide 1 receptor (GLP-1R), highlighting their activation processes upon peptide ligand binding. Comparative analysis of structures bound to endogenous and synthetic peptide ligands reveals critical insights for rational drug design. A case study on GLP-1R demonstrates how structural insights have led to the design of successful drugs for type 2 diabetes and obesity. This comparative structural analysis aims to deepen our understanding of GPCR activation mechanisms and support future drug discovery efforts targeting peptide-binding GPCRs.

## Peptide GPCRs reveal insights for targeted drug development

G protein-coupled receptors (GPCRs) constitute a large family of membrane proteins that play a key role in transmitting signals from outside to inside the cell. Developing drugs that target certain GPCRs, especially those that bind peptide ligands, is challenging due to their structural complexity. Recent advances in X-ray crystallography and cryo-electron microscopy have enabled detailed structural analyses of these receptors. The study focuses on peptide-binding GPCRs involved in critical physiological processes such as metabolism, immune response and pain perception. By examining structures of GPCRs bound to various peptides and drugs, researchers revealed conformational changes associated with receptor activation and signal transduction. These insights enhance our understanding of ligand recognition and provide valuable guidance for designing more selective and effective therapeutics targeting peptide-binding GPCRs implicated in diseases such as diabetes and cardiovascular disorders.

This summary was initially drafted using artificial intelligence, then revised and fact-checked by the author.

## Introduction

G protein-coupled receptors (GPCRs)^1^ represent the largest and most diverse superfamily of membrane proteins in human, comprising over 800 members^2^. These GPCRs are activated by a wide range of endogenous ligands, including ions, lipids, nucleotides, amines, small molecules and peptides^3^, and they play crucial roles in physiological processes, such as sensory perception^4,5^, emotional regulation^6^ and metabolic control^7,8^. Due to their extensive involvement in health and disease including cardiovascular disorders^9,10^, neurodegenerative diseases^11,12^ and metabolic syndromes^8,13^, GPCRs have emerged as prominent drug targets, with over 30% of Food and Drug Administration (FDA)-approved drugs acting on GPCRs, demonstrating their therapeutic importance^1,14^. GPCRs are classified into several families (classes A, B, C and F) based on sequence homology and domain structure^15^.

**Fig. 1:**
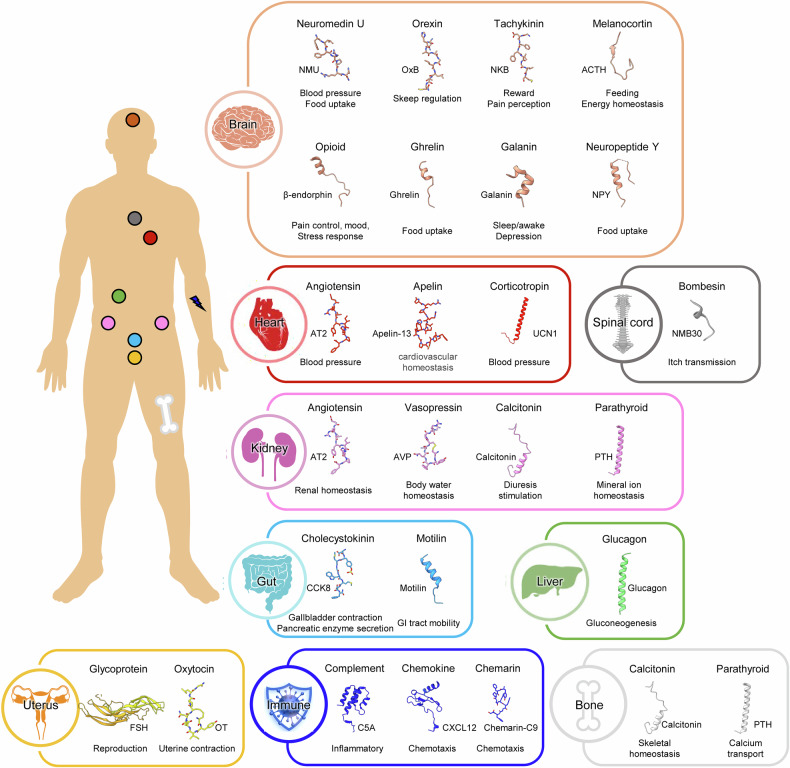
Diverse physiological roles of GPCR-targeting peptides around the human body. Representative GPCR-targeting peptides and their associated physiological roles are summarized across various regions of the human body. The structure of each peptide represents its conformation when bound to its respective GPCR (PDB: (brain) 7W53, 7L1U, 8JBG, 8GY7, 8F7Q, 7F9Y, 7XJJ and 7VGX; (heart) 6JOD, 8XZG and 6PD1; (spinal cord) 8H0P; (kidney) 7DW9, 7TYO and 8FLU; (gut) 7EZH and 8IBV; (liver) 6LML; (uterus) 8I2G and 7RYC; (immune) 8HK5, 7SK3 and 7YKD, from left to right). Peptides are shown as cartoons or stick representations.

Within this diverse family, peptide-binding GPCRs are of particular interest due to their roles in regulating key biological functions such as metabolism^8,16^, immune responses^17,18^, cardiovascular regulation^9,19,20^, energy balance^21–24^, pain perception^25,26^ and reproductive functions^27–29^. Peptide ligands, including hormonal peptides (for example, glucagon)^30^, neuropeptides (for example, endorphins)^31^ and regulatory peptides (for example, angiotensin)^32^, initiate signaling cascades by binding to their respective GPCRs, resulting in physiological responses across various organ systems (Fig. 1). The remarkable diversity of peptide ligands and their specific interactions with cognate GPCRs allow precise control of numerous physiological processes, highlighting the therapeutic potential of targeting peptide-activated GPCRs in disease management^14^.

Despite their importance for human health and disease, the development of therapeutics targeting peptide GPCRs has been challenging due to their structural flexibility and signaling complexity. However, recent advances in structural biology, particularly with X-ray crystallography and cryo-electron microscopy (cryo-EM), have revolutionized our understanding of molecular mechanisms of GPCRs’ ligand recognition and activation. Specifically, the first high-resolution crystal structures of β2-adrenergic receptor (β2AR) in both inactive and G protein-bound active states^33–35^ laid the foundation for a new era in GPCR structural biology. Since 2017, using advanced cryo-EM technology, an extensive amount of structural data on GPCR-G protein complexes has been accumulated, with approximately 950 structures (200 unique GPCR structures) reported as of October 2024. The elucidation of GPCR structures with endogenous and synthetic ligands could offer new opportunities for structure-guided drug discovery with improved selectivity and efficacy. Furthermore, these structural insights have facilitated the development of innovative pharmacological tools such as biased agonists and allosteric modulators, which offer more precise control over GPCR signaling^36^.

In this Review, we specifically highlight key peptide-activated GPCRs that are actively studied in drug development. These include class A peptide-binding GPCRs, such as angiotensin II type 1 receptor (AT1R), chemokine receptor 5 (CCR5) and μ-opioid receptor (MOR), as well as class B peptide-binding receptors such as parathyroid hormone (PTH) receptor 1 (PTH1R) and glucagon-like peptide-1 receptor (GLP-1R). By exploring their structural, pharmacological and physiological perspectives, this Review aims to offer a comprehensive understanding of peptide-activated GPCRs and their therapeutic potential. We hope these insights will inspire future research and guide drug discovery initiatives, contributing to the innovation of new therapeutics for a variety of diseases.

## Peptide-binding GPCRs: structural diversity, activation mechanism and drug development

### Structural features of peptide-binding GPCRs and types of peptide

GPCRs share a conserved seven-transmembrane (7TM) domain architecture along with three extracellular loops (ECLs), three intracellular loops (ICLs), and N-terminal and C-terminal tails of varying lengths^37^. Following TM7, most GPCRs form an intracellular α-helix (H8), which is often anchored to the membrane via lipid modification of Cys on H8^38,39^. However, certain GPCRs, such as GnIHR (gonadotropin-inhibitory hormone receptor), lack both the H8 and C-tail^40^. The lengths of ICL3 and the C-tail vary considerably, sometimes exceeding 100 residues^41^. These regions play key roles in downstream signaling, with ICL3 involved in G protein coupling and ICL3 and/or the C-tail contributing to β-arrestin binding^42,43^.

Peptide-binding GPCRs represent a substantial subset of the GPCR superfamily, recognizing a diverse range of peptide ligands that act as crucial signaling molecules in various physiological processes. Hormonal peptides, such as neuromedin U^44,45^ and ghrelin^46,47^ mediate neuroendocrine signaling and play key roles in regulating blood pressure and food intake. Neuropeptides mainly act as neurotransmitters in the central and peripheral nervous systems, influencing pain perception, emotion and behavior through their interactions with specific GPCRs. Neuropeptide Y^48,49^, galanin^50,51^, endorphins^52,53^ and orexin^54^ peptides have been extensively explored in the context of appetite regulation and pain modulation. Regulatory peptides, including angiotensin^55^, apelin^56^, endothelin^57^, vasopressin^58^ and calcitonin^59^, play essential roles in cardiovascular and body water homeostasis. In addition, chemokines^60^ and anaphylatoxins^61^, typically composed of 60–100 amino acids, are central to inflammatory responses and cell migration. To accommodate this wide range of peptide ligands—from small oligopeptides to larger polypeptide hormones—peptide-binding GPCRs exhibit structural diversity, allowing them to selectively recognize and respond to a broad spectrum of physiological signals^62,63^.

**Fig. 2:**
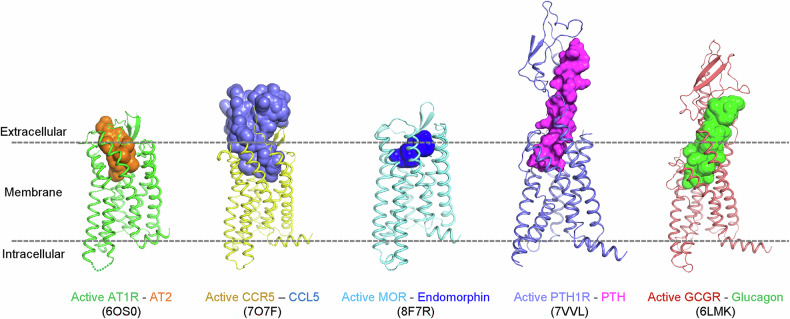
Diverse binding modes of peptide ligands to their GPCRs. Structures of five representative GPCRs in their peptide ligand-bound states are presented: AT1R (6OS0), CCR5 (7O7F), MOR (8F7R), PTH1R (7VVL), and GCGR (6LMK). Receptors are shown as cartoons in distinct colors, and G proteins (or the nanobody in PDB 6OS0) are omitted for clarity. Peptide ligands, angiotensin II (AT2), CCL5, endomorphin, PTH and glucagon, are displayed as surface models.

Recent advances in structural biology have greatly enhanced our understanding of these receptors, with about 470 peptide-bound GPCR structures determined as of 2024, including approximately 350 in the active state and 116 in the inactive state^64^. Peptide-binding GPCRs exhibit distinctive structural features, with a key characteristic being the involvement of the ECLs and the N-terminal tail in ligand binding^65^. This arrangement accommodates peptides of varying sizes and structures, distinguishing these receptors from those binding smaller molecules such as ions or amines^30,34,48,62,63,66^. Peptide-binding GPCRs exhibit structural diversity in their binding pocket architecture and ligand recognition mechanisms (Fig. 2). Some receptors have deep pockets enveloping the entire peptide, while others feature more open sites allowing peptide interaction with both transmembrane core domains and extracellular domains (ECDs)^30,32^.

### Activation mechanism of the class A/B GPCRs upon peptide ligand binding

The activation mechanism of GPCRs by diffusible ligand binding was first proposed on the basis of structural studies of β2AR^67^. Subsequent structural studies have established that ligand binding induces conformational changes within the transmembrane domain (TMD), leading to the outward movement of TM6 on the intracellular side. This movement creates a binding cavity for G protein^68^.

**Fig. 3:**
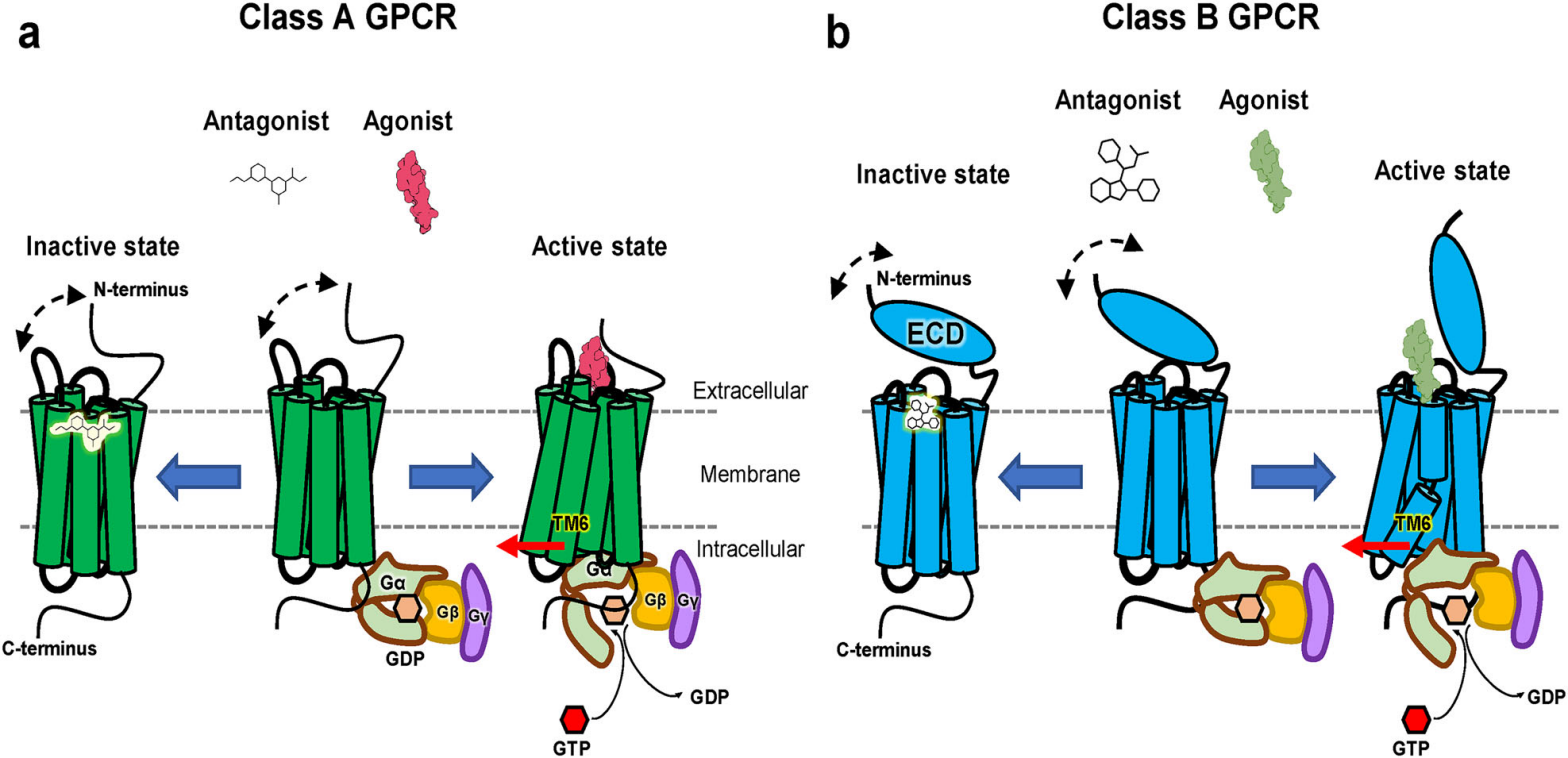
General activation mechanisms of class A and B GPCRs bound to peptide ligands. **a** A schematic representation of the inactive and active states of class A GPCRs. While small-molecule antagonists typically bind into the transmembrane (TM) pocket stabilizing the receptor in an inactive state, peptide agonists, generally, interact with ECL2 and the N-tail, which are flexible in the absence of a peptide ligand (indicated by a dashed black arrow), as well as with the TM pocket. Agonist binding induces conformational changes in the TMD, resulting in the outward movement of the cytoplasmic segments of TM5 and TM6 (indicated by a red arrow), thereby facilitating G protein binding. **b** A schematic representation of the inactive and active states of class B GPCRs. These receptors possess a large N-terminal ECD, which is flexible in the absence of a peptide ligand (dashed black arrow). Peptide agonists engage both the ECD and TMD. In the active state, a sharp kink or bend in the PxxG motif of TM6 promotes the outward movement of its cytoplasmic segment (red arrow), creating an open cavity for G protein binding.

Once activated, GPCRs interact with specific G protein subtypes (G_s_, G_i_, G_q_ and G_12/13_), each initiating distinct signaling cascades^1^. This specificity in G protein coupling contributes to the diverse functional outcomes observed across the GPCR family. For instance, G_s_ proteins stimulate adenylyl cyclase, increasing cyclic AMP production, while G_i_ proteins inhibit this enzyme^69^. G_q_ proteins activate phospholipase C, leading to calcium mobilization, and G_12/13_ proteins regulate Rho GTPases^69^. This variety in G protein-mediated pathways underlies the ability of GPCRs to modulate a wide array of cellular processes, from neurotransmission to metabolism and cell growth^13,70,71^. The structural basis for ligand binding provides crucial insights into GPCR function. Agonists typically stabilize the active conformation of the receptor, promoting G protein coupling, while antagonists stabilize the inactive state or prevent activation-associated conformational changes^72^ (Fig. 3).

Interestingly, subtle differences in ligand binding modes can lead to distinct functional outcomes, including biased signaling. In this phenomenon, different ligands can preferentially activate specific G protein pathways or β-arrestin recruitment through the same GPCR, adding another layer of complexity to GPCR signaling^73–75^. Ligands that induce biased signaling are called biased ligands, and they are regarded as an important future direction in the field of GPCR therapeutics^76^. The ability of biased ligands to preferentially engage certain downstream effectors is thought to stem from the receptor adopting distinct conformational states. In recent drug development of opioid receptor, many efforts have primarily focused on designing biased agonists to mitigate the adverse effects of traditional opioid ligand. ‘Balanced’ opioid ligands, which activate both G protein and β-arrestin pathways, have been associated with several side effects, such as respiratory dysfunction, which can be fatal^77^. By contrast, oliceridine, a G protein-biased opioid ligand, exhibits analgesic effects while minimizing adverse effects such as constipation and respiratory dysfunction^78^. Although still in its early stages, the therapeutic application of biased ligands will advance through structural analysis, enabling precise modulation of receptor signaling.

Peptide GPCRs belonging to the class A subfamily, distinguished by a relatively short N-terminal ECD compared with class B receptors, follow the canonical class A GPCR activation mechanism. They possess several conserved features crucial for their function, including the ‘DRY’ motif at the intracellular end of TM3, essential for G protein coupling and receptor activation, and the ‘NPxxY’ motif in TM7, important for receptor stability and activation^68^. A highly conserved disulfide bridge between Cys residues in ECL2 and TM3 plays a role in stabilizing the extracellular region. The binding pocket in class A GPCRs is typically deep and narrow, allowing various ligand binding orientations^68,79^. Despite these common features, considerable diversity exists in ligand recognition and the activation mechanisms among class A peptide GPCRs^79^.

By contrast, class B peptide GPCRs exhibit a distinct structural architecture featuring a large N-terminal ECD that is critical for peptide ligand recognition. This ECD, typically 120–160 amino acids long, adopts a conserved fold stabilized by three disulfide bonds, often referred to as the secretin family recognition fold^80^. The peptide binding mechanism in class B GPCRs involves a two-step process: C-terminal portion of the peptide first engages with the ECD, followed by the N-terminal portion interacting with the TMD. This ‘two-domain’ binding mode enables high-affinity and selective recognition of larger peptide hormones^81,82^. Class B GPCRs also feature a deeper and more open binding pocket in the transmembrane region compared with class A receptors. They lack the ‘DRY’ motif, which is replaced by conserved ‘HETx’ motif in TM3, and possess PxxG motif in TM6^83^. These structural differences between these two GPCR classes reflect their adaptation to different types of peptide ligands and activation mechanisms^84^.

### Structural characteristics of the peptide-binding GPCRs and structure-based drug development

Many peptide-binding GPCRs are well-known therapeutic targets^14,85^(Table 1). Structural studies of these peptide GPCRs in both their inactive and active states have greatly improved our understanding of how natural peptide ligands and synthetic drugs interact with their respective GPCRs at the molecular level. These insights are invaluable for structure-based drug developments^86^. In this section, we explore the structural features of key peptide-activated GPCRs from both class A and class B, shedding light on their molecular mechanisms and relevance to structure-based drug development. Our analysis includes AT1R, CCR5 and MOR from class A, as well as PTH1R and GLP-1R from class B. Through these case studies, we aim to illustrate the diversity among peptide-activated GPCRs and highlight their distinct molecular mechanisms and potential for drug development.

**Table 1:** List of FDA-approved drugs targeting peptide-binding GPCRs.

GPCR	Endogenous peptide ligand	Related symptom or disease	Drug name	Mechanism of action	FDA approved	Related PDB
AGTR1	Angiotensin	Hypertension	Olmesartan	Antagonist		4ZUD
		Hypotension or other vasodilatory shock	Angiotensin II	Agonist	2017	6OS0
AGTR2	Angiotensin	Hypotension or other vasodilatory shock	Angiotensin II	Agonist	2017	6JOD
C5AR1	Complement peptide	ANCA-associated vasculitis	Avacopan	Antagonist	2021	6C1R
CCR5	Chemokine	HIV infection	Maraviroc	Antagonist	2007	4MBS
EDNRB	Endothelin	Pulmonary arterial hypertension	Bosentan	Antagonist	2001	5XPR
		Acute cerebral ischemic stroke	Sovateltide	Agonist		8HBD, 6IGL
FSHR	Glycoprotein hormone	Female infertility	Follitropin beta	Agonist		8I2G
GHSR	Ghrelin	Growth hormone deficiency	Ibutamoren	Agonist		7NA8
GNRHR	Gonadotrophin-releasing hormone	Pain in endometriosis	Elagolix	Antagonist	2018	7BR3
LSHR	Glycoprotein hormone	Fertility problem	Chorionic gonadotropin	Agonist		7FII, 7FIG
MC4R	Melanocortin	Hypoactive sexual desire dysfunction	Bremelanotide	Agonist	2019	7F55
		Obesity	Setmelanotide	Agonist	2020	7PIU, 7AUE
		Phototoxicity in adults with erythropoietic protoporphyria	Afamelanotide	Agonist	2019	7PIV, 7F54
MSHR	Melanocortin	Hypoactive sexual desire dysfunction	Bremelanotide	Agonist	2019	7F4I
NK1R	Tachykinin	Nausea, vomiting	Aprepitant	Antagonist	2003	6J20, 6J21, 6HLO
		Chemotherapy-induced nausea	Netupitant (+ palonosetron)	Antagonist	2014	6HLP
OPRK	Opioid	Uremic pruritus	Nalfurafine	Agonist		7YIT
OPRM	Opioid	Analgesia, pain	Fentanyl	Agonist	1968	8EF5
		Chronic pain	Morphine	Agonist	1984	8EF6
		Pain	Oliceridine	Agonist	2020	8EFB
OX2R	Orexin	Insomnia	Suvorexant	Antagonist	2014	6TPJ, 4S0V
		Insomnia	Lemborexant	Antagonist	2019	7XRR
OXYR	Vasopressin and oxytocin	Aid in labor and delivery	Oxytocin	Agonist	1980	7QVM, 7RYC
PAR1	Proteinase-activated	Thrombotic cardiovascular events in patients with a history of myocardial infarction	Vorapaxar	Antagonist	2014	3VW7
SSR2	Somatostatin	Acromegaly	Octreotide	Agonist	1988	7YAE, 7Y26, 7Y24, 7XAU, 7T11
		Acromegaly	Lanreotide	Agonist	2007	7XAV
TRFR	Thyrotropin-releasing hormone	Diagnostic tool to test the function of thyroid gland	Protirelin (TRH)	Agonist	1976	7XW9, 7X1U, 7WKD
TSHR	Glycoprotein hormone	Hypothyroidism	Thyrotropin (TSH)	Agonist	1953	7XW5, 7UTZ, 7T9I
V2R	Vasopressin and oxytocin	Diabetes insipidus	Vasopressin	Agonist		7R0C, 7DW9, 7BB6, 7BB7, 7KH0
CALCR	Calcitonin	Osteoporosis	Salmon calcitonin	Agonist	1978	7TYN, 7TYW, 7TYY, 6NIY, 5UZ7
		Paget’s disease	Calcitonin human	Agonist	1986	7TYO, 7TYH
		Type-1/2 diabetes mellitus	Pramlintide	Agonist	2005	(Analog) 8F0J, 8F2B, 8F2A
GIPR	Glucagon	Type 2 diabetes	Tirzepatide	Agonist	2022	7VAB, 7FIY
GLP-1R	Glucagon	Type 2 diabetes	Tirzepatide	Agonist	2022	7RGP, 7VBI, 7FIM
		Type 2 diabetes	Exenatide	Agonist	2005	7LLL, 7S1M, 7S3I
		Type 2 diabetes, obesity	Semaglutide	Agonist	2017	7KI0
GLR	Glucagon	Hypoglycemia	Glucagon	Agonist	1960	8JRV, 6LMK, 6LML
PTH1R	PTH	Postmenopausal osteoporosis	Abaloparatide	Agonist	2017	7Y35, 8FLS
		Osteoporosis	Teriparatide	Agonist	1987	7Y36, 8HAO, 8HA0, 7VVK, 7VVL, 7VVN
SCTR	Secretin	Gastrinoma, pancreas dysfunction	Secretin	Agonist	2002	7D3S, 6WI9, 6WZG

Drugs that target peptide-binding GPCRs and their structural data (PDB IDs) are listed. *ANCA* antineutrophil cytoplasmic antibodies, *PDB* Protein Data Bank.

#### AT1R

The AT1R serves as a typical model for understanding the activation mechanism of peptide-binding GPCRs^55,87^, particularly those responding to hormonal peptides. AT1R is mainly involved in blood pressure regulation and is a major target for antihypertensive drugs^88^. In addition, AT1R plays important roles in fluid homeostasis^89^, cell growth^90^ and inflammatory responses^91,92^, making it relevant in various cardiovascular and renal disorders^93,94^. The structures of AT1R in both inactive and active states have provided valuable insights into its activation mechanism by the natural ligand angiotensin II^88,95^.

In the inactive state, AT1R adopts a compact conformation with the typical 7TM bundle characteristic of class A GPCRs^95^. The binding pocket is primarily formed by residues in the extracellular half of the transmembrane helices and the ECLs. A key feature of the inactive state is the presence of a major hydrogen bond network (MHN) involving residues N111^3.35^, N295^7.46^, D74^2.50^ and N298^7.49^ (superscripts denote Ballesteros–Weinstein numbering^96^), which acts as a molecular switch that stabilizes the inactive conformation. This network links TM2, TM3 and TM7, maintaining the receptor in a closed, inactive state^97^.

**Fig. 4:**
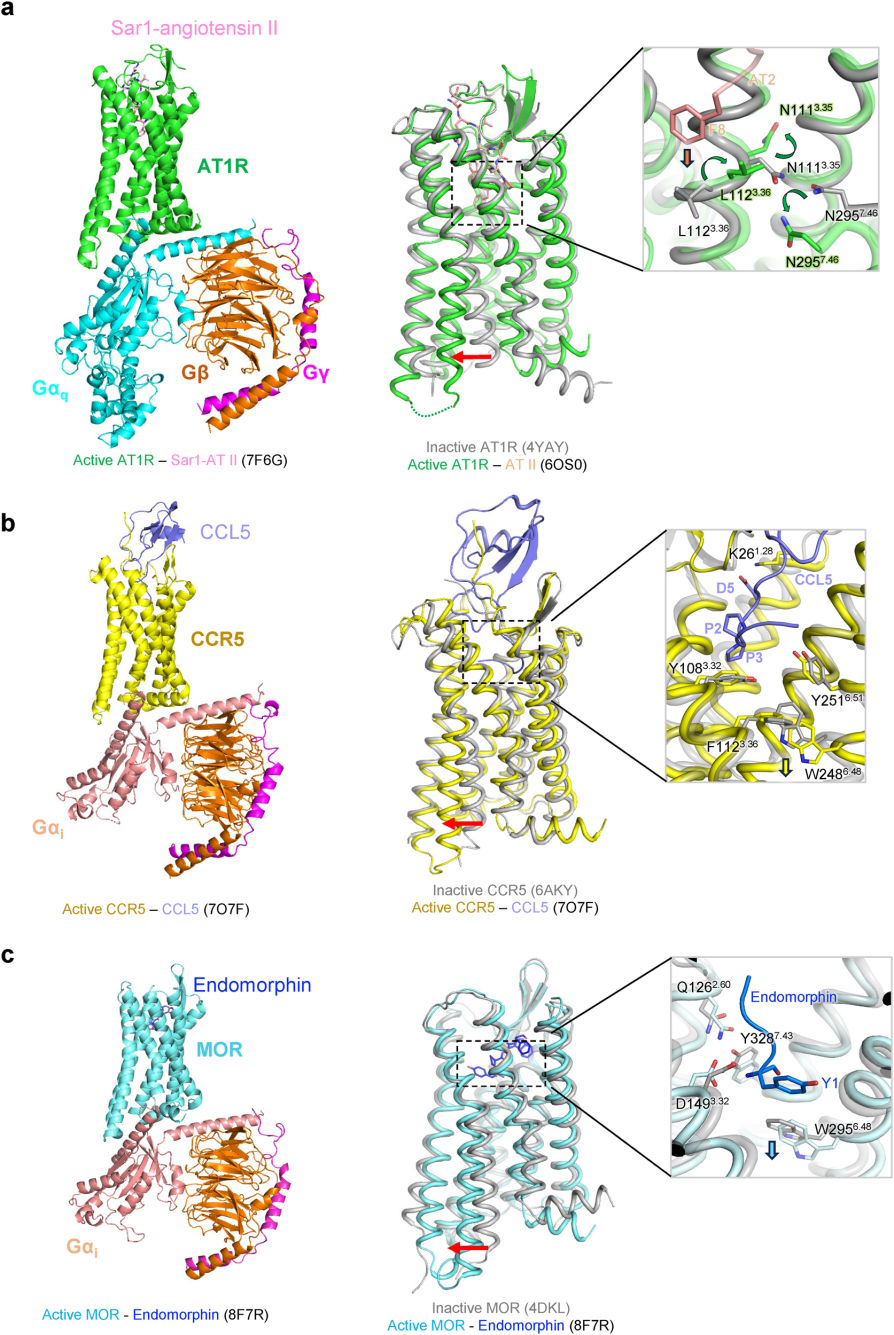
Activation mechanisms of peptide-binding class A GPCRs. **a** Left: active structure of the Sar1-angiotensin II‒AT1R‒G_q_ complex (PDB: 7F6G), color-coded as follows: Sar1-Angiotensin II (pink), AT1R (green), Gα_q_ (cyan), Gβ (orange) and Gγ (magenta). Middle: structural comparison of the active (green, PDB: 6OS0) and inactive (gray, PDB: 4YAY) states of AT1R. Right: close-up view of the binding pocket highlighting the critical role of the C-terminal phenylalanine (F8) of angiotensin II in receptor activation. Conformation changes in key residues are indicated by green arrows, while the red arrow marks the outward movement of TM6 upon activation. **b** Left: active structure of CCR5 (yellow), in complex with CCL5 (slate) and the G_i_ heterotrimer (Gα_i_ (salmon), Gβ (orange), and Gγ (magenta)) (PDB: 7O7F). Middle: structural comparison between the active (yellow, PDB: 7O7F) and inactive (gray, PDB: 6AKY) states of CCR5. Right: close-up of the orthosteric pocket showing how the N-terminal region of CCL5 interacts with the receptor. Upon activation, conformational changes in W248^6.48^ and the outward movement of TM6 are indicated by yellow and red arrows, respectively. **c** Left: active structure of MOR (cyan) in complex with endomorphin (blue) and the G_i_ heterotrimer (PDB: 8F7R). Middle: structural comparison between the active (cyan, PDB: 8F7R) and inactive (gray, PDB: 4DKL) states of MOR. Right: close-up of the orthosteric pocket showing the binding of Y1 of endomorphin (blue). The red arrow highlights the outward movement of TM6 during activation.

Upon binding of the angiotensin II peptide, substantial conformational changes occur, initiating the receptor activation process^88^. The peptide interacts extensively with residues in the ECLs and the upper portions of the transmembrane helices. The N-terminal part of angiotensin II, particularly the R2 residue, forms critical interactions with D263^6.58^ and D281^7.32^ in the extracellular region of the receptor. These interactions help position the peptide correctly in the binding pocket. The middle portion of the angiotensin II peptide makes various contacts with residues lining the binding pocket, contributing to the specificity and high affinity of the interaction. Notably, the C-terminal phenylalanine (F8) of angiotensin II plays a crucial role in receptor activation. It penetrates deep into the binding pocket, causing a conformational change in L112^3.36^ and inducing a shift and rotation of N111^3.35^. This movement disrupts the inactive state MHN, particularly breaking the N111^3.35^–N295^7.46^ hydrogen bond, which serves as a trigger for AT1R activation. The large side chain of F8 also pushes against the conserved toggle switch W253^6.48^ and Y292^7.43^, initiating conformational changes in the intracellular parts of TM6 and TM7^88^ (Fig. 4a).

In the G_q_-coupled state, the MHN undergoes extensive rearrangement. Specifically, new hydrogen bonds form between residue N294^7.45^–S252^6.47^, N294^7.45^–W253^6.48^ and D74^2.50^–N298^7.49^, stabilizing the active conformation. The α5 helix of Gα_q_ integrates into the intracellular pocket of AT1R, establishing hydrogen bonds and hydrophobic interactions with residues in TM3, TM5, TM6 and helix 8. In this AT1R–Gα_q_ complex, the α5 helix of Gα_q_ is positioned closer to TM7 and helix 8, compared with the other GPCR such as D1R–Gα_s_ and D2R–Gα_i_ structures. This positioning is stabilized by a hydrogen bond between G306^8.47^ of AT1R and N357 of Gα_q_. This structural feature provides insight into the specific interaction pattern of AT1R with its cognate G_q_ protein^97^.

This structural information, combined with functional studies, provides a detailed molecular model for AT1R activation and signaling by its natural ligand. It highlights the importance of the MHN as a central regulator of receptor conformational states and illustrates how peptide binding can induce large-scale conformational changes through a series of local perturbations. The mechanism elucidated for AT1R probably shares common features with other peptide-binding GPCRs, particularly those responding to hormonal peptides. Regarding drug development, the natural ligand angiotensin II itself is used therapeutically to treat hypotension in severe conditions such as septic shock^98–100^. In addition, drugs like olmesartan serve as inverse agonists or antagonists of AT1R, for hypertension and related cardiovascular disorders treatment^101^(Table 1). These structural insights have important implications for the design of new GPCR-targeted drugs with improved efficacy and specificity. Future research may focus on further elucidating the specifics of AT1R–G protein coupling and exploring the potential for biased agonism in drug development.

#### CCR5

CCR5, crucial for immune cell trafficking and human immunodeficiency virus (HIV) co-receptor function, exemplifies another case of peptide-activated class A GPCRs^102–104^. The primary endogenous ligands for CCR5 include CCL3 (MIP-1α), CCL4 (MIP-1β) and chemokine ligand 5 (CCL5; RANTES)^105^. Structural studies of CCR5 in ligand-free and chemokine-bound states have illuminated its activation mechanism and ligand recognition. In the inactive state, CCR5 displays the characteristic 7TM bundle of class A GPCRs with a relatively open extracellular vestibule^106,107^. The binding pocket is formed by residues from the ECLs and upper transmembrane helices, featuring several acidic residues important for recognizing basic residues in chemokine ligands^107^. Specifically, key residues Y108^3.32^, F109^3.33^, F112^3.36^, W248^6.48^ and Y251^6.51^ form a hydrophobic pocket that plays a crucial role in antagonist recognition and maintaining the receptor in an inactive conformation^106^.

The active state structure of CCR5 bound to its native ligand CCL5 (RANTES) reveals notable conformational changes. The N-terminus of CCL5 penetrates deeply into the transmembrane core of CCR5, interacting with residues in TM1, TM2, TM3 and TM7. This deep insertion is facilitated by the extended N-terminal region of CCL5, which adopts a straight conformation stabilized by interactions with a cluster of hydrophobic residues in TM2 and TM3 of CCR5. Notably, the D5 residue of CCL5 forms a crucial ionic interaction with K26^1.28^ in TM1 of CCR5, which appears to be a key determinant of agonist activity. The chemokine core domain interacts with CCR5 ECLs and the N-tail, providing additional binding energy and specificity. Activation of CCR5 involves a large outward movement of TM6, similar to other class A GPCRs, accompanied by rearrangements in the intracellular portions of other transmembrane helices, particularly TM5 and TM7^107^.

The CCL5 N-terminus, especially residues 3–5, plays a crucial role in receptor activation by pushing against key structural motifs at the bottom of the orthosteric pocket. This interaction triggers a cascade of conformational changes, including rearrangements of an aromatic connector in TM3 and TM6, as well as movements in the TM7 backbone. These changes propagate through the receptor, activating the canonical class A GPCR microswitch network, including the PIF, NPxxY and DRY motifs. Notably, the tryptophan residue W248^6.48^ acts as a toggle switch in this activation process, connecting the rearrangements initiated by the chemokine N-terminus to the large-scale relocation of TM6 and TM7^107^. This activation mechanism differs from that observed in some other chemokine receptors, such as CCR6, which are activated by chemokines with shorter N-termini through a distinct mechanism involving shallower binding and specialized sequence signatures^108^ (Fig. 4b).

In the G_i_-coupled state, the interface between CCR5 and the G protein is divided into rim and core regions. At the rim, ICL2 of CCR5 interacts with the N-terminal helix (αN) and adjacent β-strands (β2–β3) of Gα_i_, while ICL3 engages with β-strands (β4–β6) of Gα_i_. In the core, the α5 helix of Gα_i_ forms unique interactions with the cytoplasmic sides of TM2, TM3 and TM5 in CCR5. Notably, the C-terminal hook of α5 helix of Gα_i_ (residues 352–354) leans toward TM6 and C-terminal region of CCR5, creating distinctive contacts. For instance, E302^H8^ in CCR5 interacts with the α5 hook differently compared with other G_i_-coupled receptors such as neurotensin type 1 receptor^109^ or MOR^110^.

**Fig. 5:**
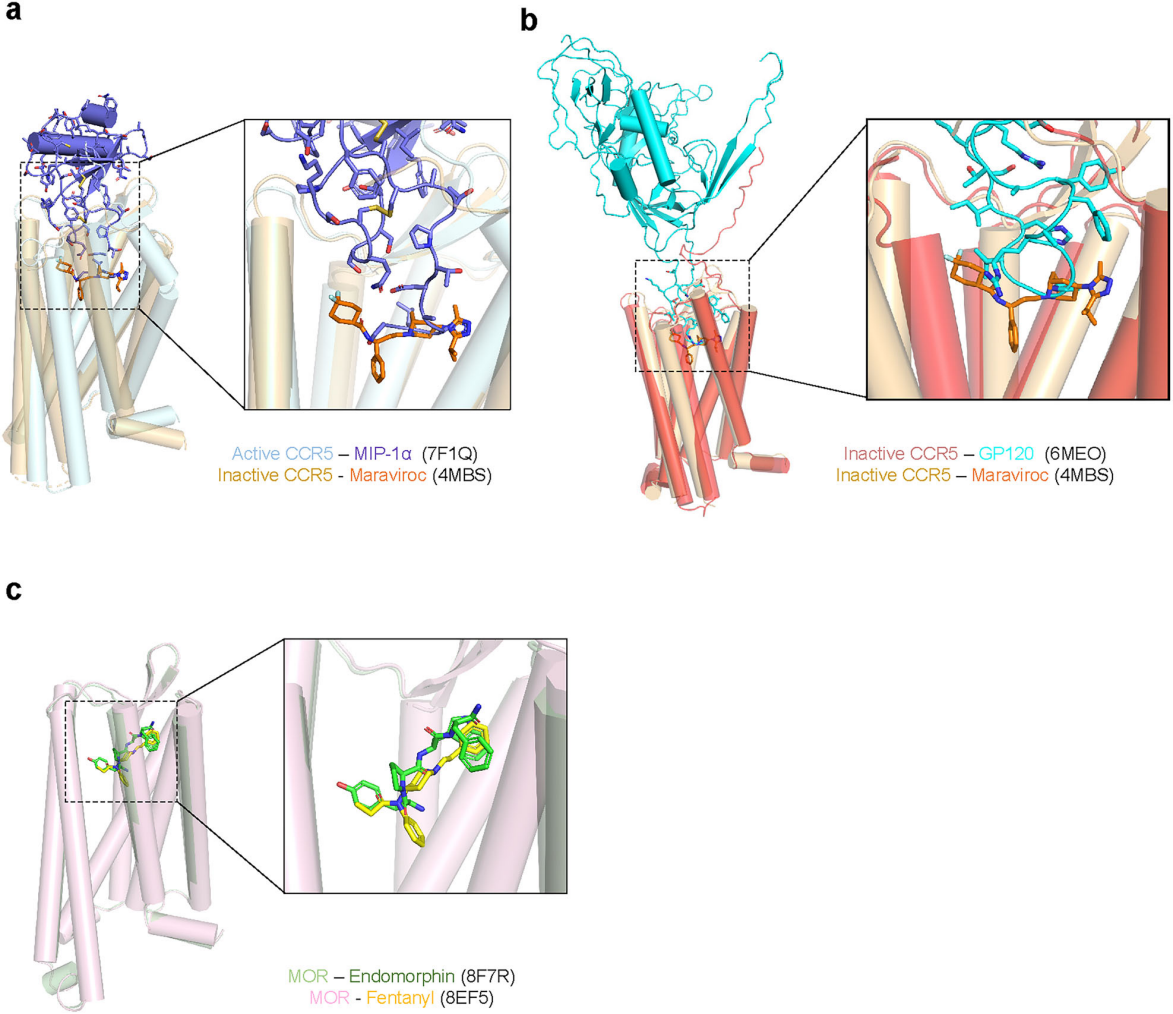
Structural comparison of endogenous and synthetic ligands bound to class A GPCRs. **a** Superposition of MIP-1α-bound active and maraviroc-bound inactive structures of CCR5. The receptor is shown as a cartoon with α-helices depicted as cylindrical helices. Ligands are shown as sticks and color-coded as follows: active CCR5 (pale cyan), MIP-1α (slate), inactive CCR5 (light orange) and maraviroc (orange). A close-up view of the TM pocket is shown on the right. The N-terminal region of MIP-1α binds deeply into the TM pocket, with maraviroc occupying a similar position. **b** Superposition of GP120-bound and maraviroc-bound inactive structures of CCR5. The receptor is shown as a cartoon and ligands are represented as sticks. GP120-bound CCR5 and GP120 are colored light red and cyan, respectively. For clarity, CD4 has been omitted. Maraviroc-bound CCR5 and maraviroc are colored light orange and orange, respectively. The binding pocket of CCR5 is highlighted in a zoomed-in view. **c** Superposition of endomorphin-bound and fentanyl-bound active structures of MOR. The receptor is shown as a cartoon, and ligands are shown as sticks, color-coded as follows: endomorphin-bound MOR (pale green), endomorphin (green), fentanyl-bound MOR (light pink) and fentanyl (yellow). A close-up of the TM pocket highlights the binding positions of the two MOR ligands.

CCR5 has been successfully targeted by the small-molecule antagonist maraviroc for HIV treatment^111^(Table 1). Structural comparison of CCR5 bound to its natural ligand MIP-1α (CCL3) and to maraviroc reveals distinct binding modes^112^ (Fig. 5a). While MIP-1α engages the orthosteric site formed by the ECLs and upper portions of the transmembrane helices, maraviroc^112^ occupies an allosteric pocket deeper within the transmembrane bundle. This binding mode prevents the conformational changes necessary for receptor activation and HIV entry (Fig. 5b). The maraviroc-bound structure shows a contraction of the ligand-binding pocket compared with the MIP-1α-bound state, with key interactions formed between the drug and residues in TM1, TM2, TM3 and TM7^112,113^ (Fig. 5a).

Following the clinical success with maraviroc, other CCR5 antagonists are in development, showing potential not only as HIV entry inhibitors but also as therapeutics for certain inflammatory diseases^106,114–118^. Future research directions include deeper understanding of CCR5’s ligand selectivity mechanisms to develop more effective and safer CCR5 modulators. Given CCR5’s diverse physiological functions, the development of biased ligands that selectively modulate specific signaling pathways could be an intriguing research topic.

#### MOR

MOR, a primary target for pain management, has been extensively studied structurally, providing valuable insights into its activation mechanism and specific ligand recognition^31,119–121^. Recent cryo-EM structures of MOR bound to endogenous opioid peptides β-endorphin and endomorphin, as well as the synthetic agonist DAMGO, have further elucidated the molecular basis of MOR activation^31,119^.

The crystal structure of MOR bound to the antagonist β-funaltrexamine provides insights into its inactive conformation. The intracellular face of MOR resembles that of rhodopsin, particularly in the relative positions of TM3, TM5 and TM6. A notable feature is the absence of an ionic bridge between the DRY motif and the cytoplasmic end of TM6, similar to the β2AR. Instead, R165^3.50^ of the DRY motif forms a salt bridge with the adjacent D164^3.49^ residue^120^.

The active state structures reveal that the peptide ligands bind in an extended conformation, with their N-termini penetrating deep into the transmembrane core. This binding mode is consistent across different peptide agonists, including DAMGO, β-endorphin and endomorphin^31,119^. The N-terminal ‘YGGF’ motif of opioid peptides occupies a conserved activation chamber at the bottom of the orthosteric binding pocket, which is crucial for receptor activation. A key interaction for receptor activation involves the tyrosine residue at the N-terminus of the peptide agonists. This tyrosine forms a network of interactions with residues D149^3.32^, Q126^2.60^ and Y328^7.43^. The primary amine group of the N-terminal tyrosine forms a salt bridge with D149^3.32^, which is stabilized by hydrogen bonds with Q126^2.60^ and Y328^7.43^. Activation of MOR involves several coordinated conformational changes. The binding of the agonist, particularly the insertion of the N-terminal tyrosine, induces a rotation of W295^6.48^ in the CWxP motif, acting as a toggle switch for activation. This leads to a large outward movement of TM6, accompanied by rearrangements in TM5 and TM7. These movements are coupled with a rearrangement of the PIF motif (P246^5.50^, I157^3.40^ and F291^6.44^), reorganization of the NPxxY motif in TM7 and restructuring of the DRY motif in TM3. Collectively, these changes create a cavity on the intracellular side of the receptor for G protein binding (Fig. 4c).

The structures also provide insights into the selectivity of different peptide agonists. While the N-terminal ‘YGGF’ motif is crucial for activation, the C-terminal sequences of the peptides interact with the extracellular regions of the receptor, contributing to binding affinity and selectivity. For instance, β-endorphin forms extensive interactions from the bottom of the binding pocket to the top extracellular regions, including residues from TM1/2/3, TM5/6/7 and ECL1/2^31^.

MOR exemplifies how structural biology can inform the development of drugs with improved safety profiles (Table 1). Comparison of MOR structures bound to the endogenous peptide endomorphin and the synthetic opioid fentanyl has revealed critical differences in their binding modes and receptor activation mechanisms. Endomorphin binds in an extended conformation with its N-terminus penetrating deep into the orthosteric pocket, forming key interactions with D149^3.32^. By contrast, fentanyl adopts a more compact binding pose, occupying a subset of the peptide binding site. Its piperidine ring mimics the tyramine moiety of endomorphin, but its unique structure allows additional contacts within the binding pocket, namely the minor pocket. These structural differences contribute to the higher potency and distinct pharmacological profile of fentanyl^31^ (Fig. 5c).

These structural studies have greatly advanced our understanding of MOR activation by endogenous and synthetic peptide agonists, providing a molecular framework for the development of novel pain medications with improved efficacy and reduced side effects. The detailed insights into the conserved activation mechanism and the specific interactions of different peptide agonists offer new opportunities for structure-based drug design targeting the opioid receptor family.

#### PTH1R

The PTH1R is a class B1 GPCR that is activated by the endogenous peptide hormones PTH and PTHrP^122^. PTH1R plays a crucial role in skeletal development and the maintenance of calcium and phosphate homeostasis^123^. Dysfunction of PTH1R has been implicated in several diseases, such as hypercalcemia^124^ and osteoporosis^125^, making it a key target for drug development.

**Fig. 6:**
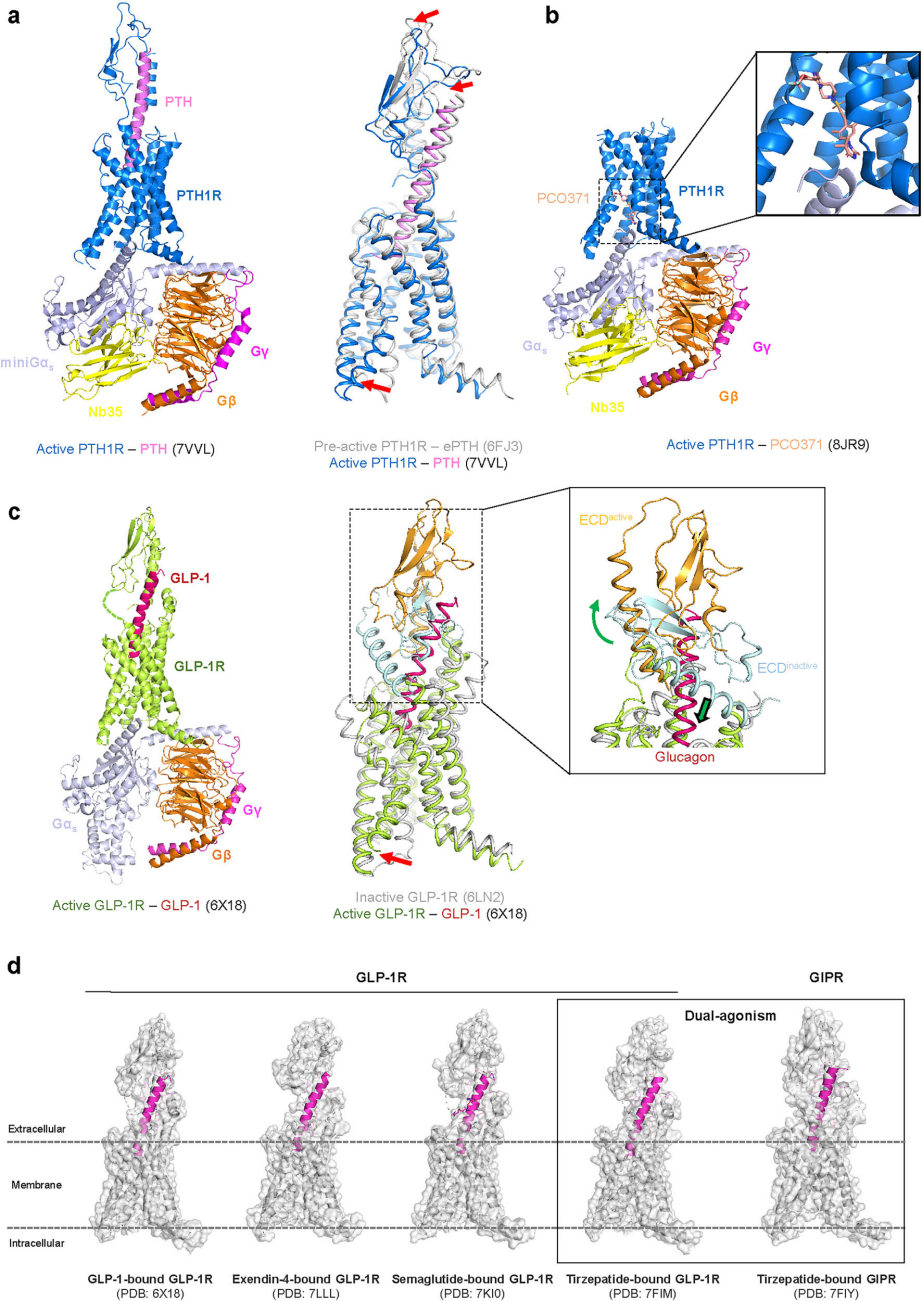
Activation and therapeutic targeting of peptide-binding class B GPCRs. **a** The active structure of the PTH‒PTH1R‒G_s_ complex is presented, with components color-coded as follows: PTH (violet), PTH1R (blue), Gα_s_ (blue white), Gβ (orange), Gγ (magenta) and Nb35 (yellow). A structural comparison of the preactive and active states of PTH1R is shown on the right. The G protein is omitted for clarity. The conformational changes from preactive to active states are indicated with red arrows. **b** The active structure of PTH1R bound to PCO371 is shown. PCO371 binds to an intracellular allosteric site, distinct from the orthosteric peptide-binding pocket. PCO371 is shown as salmon-colored sticks. A zoomed-in view on the right highlights the novel binding site of PCO371. **c** The active structure of GLP-1R (lime) bond to GLP-1 (warm pink) is shown on the left. Superposition of the active and inactive GLP-1R structures is shown on the right (G protein omitted for clarity). ECD, which undergoes dramatic rotation upon GLP-1 binding, is shown in orange (active) and pale cyan (inactive), and the movements of the ECD and TM6 upon activation are indicated with green and red arrows, respectively. **d** Structures of GLP-1R bound to endogenous ligand GLP-1 and various agonist drugs are presented. A comparison of GLP-1R and GIPR structures, both bound to the dual-agonist tirzepatide, is shown on the right. Receptors are displayed as transparent surfaces with light-gray-colored cartoons, and ligands are shown as magenta cartoons.

Structurally, PTH1R consists of a large N-terminal ECD and a TMD. The two-step binding model describes the association between the ECD and the C-terminus of the peptide ligand, as well as the interaction between the TMD and the N-terminus of the ligand. This sequential interaction is a hallmark of receptor activation in class B GPCRs. A structural comparison of engineered PTH (ePTH)-bound PTH1R and PTH-bound PTH1R reveals conformational changes upon receptor activation^126,127^. Upon activation, TM1, TM2, TM7, the ECD and the ligand move toward TM5, allowing V2^PTH^ and I5^PTH^ to access the hydrophobic cluster within the receptor. This movement creates a steric clash between PTH and the receptor, resulting in the unwinding and repositioning of TM6. TM6 movement then leads to the formation of a hydrogen bond between P415^6.47^, Y421^6.53^ and Q451^7.49^. This ‘PYQ motif’ is highly conserved within class B GPCRs and is important for receptor activation. Meanwhile, activation disrupts another highly conserved ‘HETY motif’, which is essential for maintaining the inactive conformation of PTH1R. The collapse of this motif induces a kink in TM6, resulting in the opening of the intracellular cavity to accommodate Gα_s_ binding. In the active state, the α5 helix of Gα_s_ stabilizes the receptor–G protein complex by forming hydrophobic and water-mediated interactions (Fig. 6a).

Therapeutic strategies targeting PTH1R have traditionally focused on synthetic peptides or peptide analogs of PTH and PTHrP, mimicking the action of endogenous ligands^128^. However, these peptide therapies require injections and are associated with adverse effects such as bone resorption^129^. To address these limitations, small-molecule agonists have been developed as alternative modulators of PTH1R signaling. One such example is PCO371, a G protein-biased agonist of PTH1R that can be administered orally^130^. Cryo-EM structural analysis revealed that PCO371 binds to an intracellular allosteric pocket, distinct from the orthosteric PTH binding site, where it interacts directly with the C-terminus of Gα_s_^131,132^ (Fig. 6b). This unique binding mode contributes to biased signaling, stabilizing the receptor–G protein complex while reducing β-arrestin recruitment.

Structural studies of PTH1R have provided key insights into its activation mechanism and ligand recognition. The discovery of PCO371, a nonpeptide G protein-biased agonist that binds to an intracellular allosteric site, highlights the potential for small-molecule modulation of peptide-binding GPCRs. Further exploration of allosteric modulators and biased ligands will be essential for advancing GPCR-targeted drug discovery and expanding therapeutic options.

### GLP-1R

GLP-1R is a class B1 GPCR that has emerged as a crucial therapeutic target for type 2 diabetes and obesity treatment^133,134^. GLP-1R plays a pivotal role in glucose homeostasis by enhancing glucose-dependent insulin secretion^135^, suppressing glucagon release^136^ and promoting satiety^137,138^.

Inactive GLP-1R structure reveals a compact conformation with the ECD positioned close to the TMD. Key features include a hydrophobic interface between the ECD and TMD, and a closed conformation of the peptide-binding pocket. Residues such as E127 in the ECD and Q211 in ECL1 contribute to maintaining this inactive state through interactions between the ECD and ECL1/3. The α-helical ECL1 partially occupies the orthosteric ECD-binding site, while a segment of the ECD (residues 33–40) interacts with ECL3, further stabilizing the closed conformation^139^.

GLP-1R activation involves complex interactions between the GLP-1 peptide ligand, ECD and TMD. Crystal structures of the GLP-1R ECD bound to GLP-1 show a conserved α-helical binding mode^140^. In this structure, the N-terminus of peptide is expected to interact with the receptor TMD, while its C-terminal α-helix binds to the ECD^140^. Recent cryo-EM structures of full-length GLP-1R in complex with various ligands and G proteins have elucidated the activation mechanism^141–144^. Upon GLP-1 binding, substantial conformational changes occur. The ECD undergoes a large movement, rotating away from the transmembrane core. This allows the peptide’s N-terminus to insert deeply into the transmembrane bundle. Concurrently, TM6 moves outward by approximately 18 Å, creating a cavity for G protein binding^145^. Key residues in this process include R190^2.60^, forming a polar interaction with the E9^GLP-1^, and Q211 and H212 on ECL1 interacting with W31^GLP-1^ on the C-terminus of GLP-1. In addition, R299 on ECL2 plays an important role in stabilizing GLP-1 binding through polar interaction with H7^GLP-1^, contributing to receptor activation^145^. The ECD’s movement is crucial for exposing the peptide-binding pocket and facilitating these interactions (Fig. 6c).

Intriguingly, recent studies reveal that GLP-1R can adopt a distinct conformation when bound to G_s_ protein without an agonist^146^. This ligand-free state shows a partially activated receptor with TM6 moving outward, but the ECD and peptide-binding pocket differ from the fully active state. ECL3 adopts a more open conformation, suggesting a ‘G_s_ protein-first binding’ mechanism that may facilitate rapid signal transduction upon subsequent agonist binding^146^.

The therapeutic potential of GLP-1R has led to the development of several agonists (Table 1). Exenatide, the first GLP-1R agonist approved for clinical use, is derived from Gila monster venom. Its extended C-terminal region forms additional interactions with the ECD, contributing to higher affinity and prolonged action compared with native GLP-1^147,148^. Liraglutide, another early GLP-1R agonist, incorporates a fatty acid moiety that enhances albumin binding, extending its half-life^149,150^. Semaglutide, a more recent GLP-1 analog (marketed as Ozempic for diabetes and Wegovy for obesity), further improves upon this design^151^. Its fatty acid modification not only extends half-life but also allows for once-weekly dosing, markedly improving patient compliance^152^. Structural studies show that, while semaglutide binds similarly to GLP-1, it exhibits different receptor–peptide dynamics, particularly in the interaction between the fatty acid chain and the receptor’s surface, which may contribute to its improved pharmacological profile^153^ (Fig. 6d).

Recent developments have led to multi-target approaches. Tirzepatide, a dual GIP and GLP-1 receptor agonist, combines structural elements of both peptides^154^. Cryo-EM structures reveal how tirzepatide achieves dual agonism through a unique binding mode that allows it to engage both receptors with high potency^143^. This dual action offers enhanced efficacy in treating metabolic disorders by simultaneously improving insulin sensitivity and promoting weight loss, effects not fully achieved by GLP-1R agonism alone^143^. Ongoing research is exploring triagonists targeting GLP-1R, GIPR and GCGR simultaneously^86,155^. These molecules aim to leverage the complementary effects of all three receptors, potentially offering superior glycemic control and weight loss. Structural insights are crucial for developing these complex agonists, helping to balance receptor activation and manage potential side effects^143,156^ (Fig. 6d).

As our understanding of GLP-1R structure and activation mechanisms grows, we anticipate more targeted and efficient therapies. Future developments may include novel peptide modifications, small-molecule agonists or bispecific antibodies^157^. The discovery of ligand-free, G protein-coupled states opens new avenues for drug design, potentially enabling the development of compounds that can modulate receptor activity through novel mechanisms.

## Conclusions and future perspective

The field of structural biology for peptide-activated GPCR signaling has advanced substantially, providing crucial insights into the molecular mechanisms of peptide ligand recognition and receptor activation. Recent discoveries have profoundly influenced drug discovery, revealing both common activation themes and receptor-specific features that contribute to ligand selectivity and signaling outcomes. Such structural insights have revolutionized drug development strategies, enabling rational modification of peptide ligands and the exploration of new allosteric modulation sites. The development of long-acting GLP-1R agonists for the treatment of type 2 diabetes and obesity exemplifies this progress. Case studies of AT1R, CCR5 and MOR demonstrate how structural information guides the development of both peptide-based and small-molecule therapeutics. For example, the structures of OX1R and OX2R enabled structure-based drug design starting from suvorexant, a nonselective antagonist approved by the FDA for the treatment of insomnia^158,159^. By leveraging the distinct feature of the orthosteric pocket in the two receptor subtypes—specifically the A/T^3.33^ difference in TM3—the development of the OX1R-selective antagonist JH112 was successfully achieved, which is expected to have reduced off-target effect^160^. Despite these advances, challenges remain in structure-based drug design for peptide-binding GPCRs. The dynamic nature of these receptors and the conformational flexibility of peptide ligands complicate rational design. In addition, improving the oral bioavailability and extending the plasma half-life of peptide drugs remain major hurdles.

As of 2024, 750 class A GPCR structures and 120 class B GPCR structures have been determined, with over 120 GPCRs identified as drug development targets in IUPHAR^161^. While peptide-based therapeutics have been widely developed for class B GPCRs such as GLP-1R and PTH1R, the progress in small-molecule or orally administered drugs remains limited. Recent studies on allosteric binding sites and biased signaling mechanisms in these receptors suggest new opportunities for drug development. Expanding the application of structure-based approaches to design allosteric modulators and biased ligands will be crucial for enhancing the therapeutic potential of peptide-binding GPCRs.

Recent advancements in antibody drugs targeting GPCRs, such as erenumab, which targets the CGRP receptor for migraine treatment^162,163^, further demonstrate the evolving landscape of GPCR-targeted therapies. Antibody drugs offer advantages over small-molecule drugs, including higher selectivity, stronger binding affinity and longer persistence in the body. However, they face limitations in terms of cost-effectiveness, as they require high production costs and have administration restrictions. Moreover, their large molecular size limits blood–brain barrier penetration, which can be either an advantage or a challenge depending on the therapeutic context^164^. Erenumab has been shown to exhibit minimal blood–brain barrier penetration under normal physiological conditions. This feature is generally advantageous for avoiding central nervous system-related side effects^165,166^. Advances in bispecific antibody technology offer new strategies for improving efficacy. Talquetamab, for instance, is a bispecific antibody targeting GPRC5D (a class C GPCR) and CD3, effectively engaging T cells to eliminate multiple myeloma cells^167^. While most class A receptors, such as β2AR, lack large ECDs that facilitate antibody binding, peptide-binding class A GPCRs possess relatively long and accessible ECLs involved in ligand binding. These ECLs, which usually exhibit conformational dynamics depending on the presence or absence of peptide ligands, can be targeted by conformation-selective antibodies or nanobodies. This expands the range of GPCRs that can be targeted by antibody-based therapies^168^. With the growing availability of high-resolution structural data of peptide-bound class A GPCRs, the development of antibody drugs targeting these GPCRs is expected to accelerate, opening new avenues for therapeutic intervention.

Looking forward, cryo-EM is expected to play an increasingly important role in elucidating GPCR structures in various states. The integration of structural biology with computational modeling, medicinal chemistry and high-throughput screening promises to facilitate the discovery of novel therapeutics. Breakthrough computational tools such as AlphaFold3^169,170^ and RoseTTAFold All-Atom^171^ have opened new opportunities for GPCR-targeted drug discovery, greatly advancing structure-based drug development. These methods enable high-confidence GPCR structure prediction, ligand design and validation, thereby broadening the scope of drug discovery. While the application of cryo-electron tomography (cryo-ET) in GPCR research remains limited, this technique continues to be utilized for resolving the architecture of proteins in their native environments^172^. The small size of GPCRs, generally below 100 kDa, has posed challenges for cryo-ET application. However, the systematic diversity of GPCRs, including high-order oligomerization, underscores the potential of cryo-ET in this field^173^. Collectively, these advanced methodologies are expected to expand the therapeutic landscape of GPCR-targeted drug development. As our structural knowledge expands, we anticipate the development of more sophisticated and effective drugs targeting peptide-binding GPCRs for a wide range of diseases. The future of drug discovery in this field is promising, with potential for creating more personalized and precisely targeted therapies based on our growing understanding of GPCR function at the molecular level. These advancements may lead to new treatments for metabolic disorders, inflammatory diseases and neurological conditions.

## References

[1] Zhang, M. Y., Chen, T., Lu, X., Lan, X. B., Chen, Z. Q. & Lu, S. Y. G protein-coupled receptors (GPCRs): advances in structures, mechanisms, and drug discovery. *Signal Transduct. Target. Ther.* **9**, 88 (2024) 10.1038/s41392-024-01803-6 PMC11004190 38594257

[2] Stevens, R. C. et al. The GPCR Network: a large-scale collaboration to determine human GPCR structure and function. *Nat. Rev. Drug Discov.* **12**, 25–34 (2013). 23237917 10.1038/nrd3859 PMC3723354

[3] Sutkeviciute, I. & Vilardaga, J. P. Structural insights into emergent signaling modes of G protein-coupled receptors. *J. Biol. Chem.* **295**, 11626–11642 (2020). 32571882 10.1074/jbc.REV120.009348 PMC7450137

[4] DeMaria, S. & Ngai, J. The cell biology of smell. *J. Cell Biol.* **191**, 443–452 (2010). 21041441 10.1083/jcb.201008163 PMC3003330

[5] Roper, S. D. & Chaudhari, N. Taste buds: cells, signals and synapses. *Nat. Rev. Neurosci.* **18**, 485–497 (2017). 28655883 10.1038/nrn.2017.68 PMC5958546

[6] Grammatopoulos, D. K. Regulation of G-protein coupled receptor signalling underpinning neurobiology of mood disorders and depression. *Mol. Cell Endocrinol.* **449**, 82–89 (2017). 28229904 10.1016/j.mce.2017.02.013

[7] Barella, L. F., Jain, S., Kimura, T. & Pydi, S. P. Metabolic roles of G protein-coupled receptor signaling in obesity and type 2 diabetes. *FEBS J.* **288**, 2622–2644 (2021). 33682344 10.1111/febs.15800

[8] Jin, C., Chen, H., Xie, L., Zhou, Y., Liu, L. L. & Wu, J. GPCRs involved in metabolic diseases: pharmacotherapeutic development updates. *Acta Pharm. Sin.* **45**, 1321–1336 (2024). 10.1038/s41401-023-01215-2 PMC11192902 38326623

[9] Li, Y., Li, B., Chen, W. D. & Wang, Y. D. Role of G-protein coupled receptors in cardiovascular diseases. *Front. Cardiovasc. Med.* **10**, 1130312 (2023). 37342437 10.3389/fcvm.2023.1130312 PMC10277692

[10] Wang, J., Gareri, C. & Rockman, H. A. G-protein-coupled receptors in heart disease. *Circ. Res.* **123**, 716–735 (2018). 30355236 10.1161/CIRCRESAHA.118.311403 PMC6205195

[11] Wong, T. S. et al. G protein-coupled receptors in neurodegenerative diseases and psychiatric disorders. *Signal Transduct. Target. Ther.* **8**, 177 (2023). 37137892 10.1038/s41392-023-01427-2 PMC10154768

[12] Huang, Y., Todd, N. & Thathiah, A. The role of GPCRs in neurodegenerative diseases: avenues for therapeutic intervention. *Curr. Opin. Pharm.* **32**, 96–110 (2017). 10.1016/j.coph.2017.02.001 28288370

[13] Barella, L. F., Jain, S. & Pydi, S. P. G protein-coupled receptors: role in metabolic disorders. *Front. Endocrinol.* **13**, 984253 (2022). 10.3389/fendo.2022.984253 PMC9386565 35992121

[14] Bellmann-Sickert, K. & Beck-Sickinger, A. G. Peptide drugs to target G protein-coupled receptors. *Trends Pharm. Sci.* **31**, 434–441 (2010). 20655603 10.1016/j.tips.2010.06.003

[15] Bockaert, J., & Pin, J. P. Molecular tinkering of G protein-coupled receptors: an evolutionary success. *EMBO J.* **18**, 1723–1729 (1999). 10202136 10.1093/emboj/18.7.1723 PMC1171258

[16] Oliveira de Souza, C., Sun, X. & Oh, D. Metabolic functions of G protein-coupled receptors and beta-arrestin-mediated signaling pathways in the pathophysiology of type 2 diabetes and obesity. *Front. Endocrinol.* **12**, 715877 (2021). 10.3389/fendo.2021.715877 PMC8419444 34497585

[17] Morgan, B. P. & Harris, C. L. Complement, a target for therapy in inflammatory and degenerative diseases. *Nat. Rev. Drug Discov.* **14**, 857–877 (2015). 26493766 10.1038/nrd4657 PMC7098197

[18] Tsioumpekou, M., Krijgsman, D., Leusen, J. H. W. & Olofsen, P. A. The role of cytokines in neutrophil development, tissue homing, function and plasticity in health and disease. *Cells* **12**, 1981 (2023). 10.3390/cells12151981 PMC10417597 37566060

[19] Kaur, G., Verma, S. K., Singh, D. & Singh, N. K. Role of G-proteins and GPCRs in cardiovascular pathologies. *Bioengineering* **10**, 76 (2023). 10.3390/bioengineering10010076 PMC9854459 36671648

[20] Capote, L. A., Mendez Perez, R. & Lymperopoulos, A. GPCR signaling and cardiac function. *Eur. J. Pharm.* **763**, 143–148 (2015). 10.1016/j.ejphar.2015.05.019 25981298

[21] Lang, R. et al. Physiology, signaling, and pharmacology of galanin peptides and receptors: three decades of emerging diversity. *Pharm. Rev.* **67**, 118–175 (2015). 25428932 10.1124/pr.112.006536

[22] Jiang, G. Q. & Zhang, B. B. Glucagon and regulation of glucose metabolism. *Am. J. Physiol. Endocrinol. Metab.* **284**, E671–E678 (2003). 12626323 10.1152/ajpendo.00492.2002

[23] Tao, Y. X., Yuan, Z. H. & Xie, J. G protein-coupled receptors as regulators of energy homeostasis. *Prog. Mol. Biol. Transl. Sci.* **114**, 1–43 (2013). 23317781 10.1016/B978-0-12-386933-3.00001-7

[24] Al-Massadi, O. et al. Multifaceted actions of melanin-concentrating hormone on mammalian energy homeostasis. *Nat. Rev. Endocrinol.* **17**, 745–755 (2021). 34608277 10.1038/s41574-021-00559-1

[25] Torres, R. et al. Mice genetically deficient in neuromedin U receptor 2, but not neuromedin U receptor 1, have impaired nociceptive responses. *Pain* **130**, 267–278 (2007). 17379411 10.1016/j.pain.2007.01.036

[26] Xiao, J. et al. Neurokinin 1 and opioid receptors: relationships and interactions in nervous system. *Transl. Perioper. Pain. Med* **1**, 11–21 (2016). 28409174 PMC5388438

[27] Oduwole, O. O., Huhtaniemi, I. T. & Misrahi, M. The roles of luteinizing hormone, follicle-stimulating hormone and testosterone in spermatogenesis and folliculogenesis revisited. *Int. J. Mol. Sci.* **22**, 12735 (2021). 10.3390/ijms222312735 PMC8658012 34884539

[28] Tng, E. L. Kisspeptin signalling and its roles in humans. *Singap. Med. J.* **56**, 649–656 (2015). 10.11622/smedj.2015183 PMC4678402 26702158

[29] Foulkes, N. S., Schlotter, F., Pevet, P. & Sassonecorsi, P. Pituitary-hormone Fsh directs the crem functional switch during spermatogenesis. *Nature* **362**, 264–267 (1993). 7681549 10.1038/362264a0

[30] Qiao, A. et al. Structural basis of G_s_ and G_i_ recognition by the human glucagon receptor. *Science* **367**, 1346–1352 (2020). 32193322 10.1126/science.aaz5346

[31] Wang, Y. et al. Structures of the entire human opioid receptor family. *Cell* **186**, 413–427 (2023). 10.1016/j.cell.2022.12.026 36638794

[32] Asada, H. et al. The crystal structure of angiotensin II type 2 receptor with endogenous peptide hormone. *Structure* **28**, 418–425 (2020). 10.1016/j.str.2019.12.003 31899086

[33] Cherezov, V. et al. High-resolution crystal structure of an engineered human β2-adrenergic G protein-coupled receptor. *Science* **318**, 1258–1265 (2007). 17962520 10.1126/science.1150577 PMC2583103

[34] Rasmussen, S. G. et al. Crystal structure of the β2 adrenergic receptor–Gs protein complex. *Nature* **477**, 549–555 (2011). 21772288 10.1038/nature10361 PMC3184188

[35] Rasmussen, S. G. et al. Crystal structure of the human β2 adrenergic G-protein-coupled receptor. *Nature* **450**, 383–387 (2007). 17952055 10.1038/nature06325

[36] Kise, R. & Inoue, A. GPCR signaling bias: an emerging framework for opioid drug development. *J. Biochem.* **175**, 367–376 (2024). 38308136 10.1093/jb/mvae013

[37] Latorraca, N. R., Venkatakrishnan, A. J. & Dror, R. O. GPCR dynamics: structures in motion. *Chem. Rev.* **117**, 139–155 (2017). 27622975 10.1021/acs.chemrev.6b00177

[38] Huynh, J., Thomas, W. G., Aguilar, M. I. & Pattenden, L. K. Role of helix 8 in G protein-coupled receptors based on structure-function studies on the type 1 angiotensin receptor. *Mol. Cell Endocrinol.* **302**, 118–127 (2009). 19418628 10.1016/j.mce.2009.01.002

[39] Thibeault, P. E. & Ramachandran, R. Role of the helix-8 and C-terminal tail in regulating proteinase activated receptor 2 signaling. *ACS Pharm. Transl. Sci.* **3**, 868–882 (2020). 10.1021/acsptsci.0c00039 PMC7551709 33073187

[40] Willars, G. B. et al. Lack of a C-terminal tail in the mammalian gonadotropin-releasing hormone receptor confers resistance to agonist-dependent phosphorylation and rapid desensitization. *J. Biol. Chem.* **274**, 30146–30153 (1999). 10514504 10.1074/jbc.274.42.30146

[41] Sadler, F., Ma, N., Ritt, M., Sharma, Y., Vaidehi, N. & Sivaramakrishnan, S. Autoregulation of GPCR signalling through the third intracellular loop. *Nature* **615**, 734–741 (2023). 36890236 10.1038/s41586-023-05789-z PMC10033409

[42] Bous, J. et al. Structure of the vasopressin hormone–V2 receptor–β-arrestin1 ternary complex. *Sci. Adv.* **8**, eabo7761 (2022). 36054364 10.1126/sciadv.abo7761 PMC10866553

[43] Huang, W. et al. Structure of the neurotensin receptor 1 in complex with β-arrestin 1. *Nature* **579**, 303–308 (2020). 31945771 10.1038/s41586-020-1953-1 PMC7100716

[44] You, C. et al. Structural insights into the peptide selectivity and activation of human neuromedin U receptors. *Nat. Commun.* **13**, 2045 (2022). 35440625 10.1038/s41467-022-29683-w PMC9019041

[45] Tanaka, A. et al. Transnasal delivery of the peptide agonist specific to neuromedin-U receptor 2 to the brain for the treatment of obesity. *Mol. Pharm.* **17**, 32–39 (2020). 31765157 10.1021/acs.molpharmaceut.9b00571

[46] Sun, Y. X., Wang, P., Zheng, H. & Smith, R. G. Ghrelin stimulation of growth hormone release and appetite is mediated through the growth hormone secretagogue receptor. *Proc. Natl. Acad. Sci. USA* **101**, 4679–4684 (2004). 15070777 10.1073/pnas.0305930101 PMC384806

[47] Mani, B. K. & Zigman, J. M. Ghrelin as a survival hormone. *Trends Endocrinol. Metab.* **28**, 843–854 (2017). 29097101 10.1016/j.tem.2017.10.001 PMC5777178

[48] Park, C. et al. Structural basis of neuropeptide Y signaling through Y1 receptor. *Nat. Commun.* **13**, 853 (2022). 35165283 10.1038/s41467-022-28510-6 PMC8844075

[49] Kang, H. et al. Structural basis for Y2 receptor-mediated neuropeptide Y and peptide YY signaling. *Structure* **31**, 44–57 (2023). 36525977 10.1016/j.str.2022.11.010

[50] Kyrkouli, S. E., Strubbe, J. H. & Scheurink, A. J. Galanin in the PVN increases nutrient intake and changes peripheral hormone levels in the rat. *Physiol. Behav.* **89**, 103–109 (2006). 16806319 10.1016/j.physbeh.2006.05.009

[51] Kyrkouli, S. E., Stanley, B. G. & Leibowitz, S. F. Galanin: stimulation of feeding induced by medial hypothalamic injection of this novel peptide. *Eur. J. Pharm.* **122**, 159–160 (1986). 10.1016/0014-2999(86)90175-5 2420618

[52] Toubia, T. & Khalife, T. The endogenous opioid system: role and dysfunction caused by opioid therapy. *Clin. Obstet. Gynecol.* **62**, 3–10 (2019). 30398979 10.1097/GRF.0000000000000409

[53] Benarroch, E. E. Endogenous opioid systems: current concepts and clinical correlations. *Neurology* **79**, 807–814 (2012). 22915176 10.1212/WNL.0b013e3182662098

[54] Sakurai, T. The neural circuit of orexin (hypocretin): maintaining sleep and wakefulness. *Nat. Rev. Neurosci.* **8**, 171–181 (2007). 17299454 10.1038/nrn2092

[55] Mehta, P. K. & Griendling, K. K. Angiotensin II cell signaling: physiological and pathological effects in the cardiovascular system. *Am. J. Physiol. Cell Physiol.* **292**, C82–C97 (2007). 16870827 10.1152/ajpcell.00287.2006

[56] Wysocka, M. B., Pietraszek-Gremplewicz, K. & Nowak, D. The role of apelin in cardiovascular diseases, obesity and cancer. *Front. Physiol.* **9**, 557 (2018). 10.3389/fphys.2018.00557 PMC5974534 29875677

[57] Kohan, D. E., Rossi, N. F., Inscho, E. W. & Pollock, D. M. Regulation of blood pressure and salt homeostasis by endothelin. *Physiol. Rev.* **91**, 1–77 (2011). 21248162 10.1152/physrev.00060.2009 PMC3236687

[58] Treschan, T. A. & Peters, J. The vasopressin system—physiology and clinical strategies. *Anesthesiology* **105**, 599–612 (2006). 16931995 10.1097/00000542-200609000-00026

[59] Masi, L. & Brandi, M. L. Calcitonin and calcitonin receptors. *Clin. Cases Min. Bone Metab.* **4**, 117–122 (2007). PMC2781237 22461211

[60] Tiberio, L., Del Prete, A., Schioppa, T., Sozio, F., Bosisio, D. & Sozzani, S. Chemokine and chemotactic signals in dendritic cell migration. *Cell Mol. Immunol.* **15**, 346–352 (2018). 29563613 10.1038/s41423-018-0005-3 PMC6052805

[61] Klos, A., Tenner, A. J., Johswich, K. O., Ager, R. R., Reis, E. S. & Kohl, J. The role of the anaphylatoxins in health and disease. *Mol. Immunol.* **46**, 2753–2766 (2009). 19477527 10.1016/j.molimm.2009.04.027 PMC2725201

[62] Wang, J. et al. Cryo-EM structure of the human chemerin receptor 1–Gi protein complex bound to the C-terminal nonapeptide of chemerin. *Proc. Natl. Acad. Sci. USA* **120**, e2214324120 (2023). 36881626 10.1073/pnas.2214324120 PMC10089180

[63] Duan, J. et al. Mechanism of hormone and allosteric agonist mediated activation of follicle stimulating hormone receptor. *Nat. Commun.* **14**, 519 (2023). 36720854 10.1038/s41467-023-36170-3 PMC9889800

[64] Isberg, V. et al. GPCRdb: an information system for G protein-coupled receptors. *Nucleic Acids Res.* **44**, D356–D364 (2016). 26582914 10.1093/nar/gkv1178 PMC4702843

[65] Shihoya, W., Iwama, A., Sano, F. K. & Nureki, O. Cryo-EM advances in GPCR structure determination. *J. Biochem.* **176**, 1–10 (2024). 38498911 10.1093/jb/mvae029

[66] Wang, Y. et al. Revealing the signaling of complement receptors C3aR and C5aR1 by anaphylatoxins. *Nat. Chem. Biol.* **19**, 1351–1360 (2023). 37169960 10.1038/s41589-023-01339-w

[67] Bang, I. & Choi, H. J. Structural features of β2 adrenergic receptor: crystal structures and beyond. *Mol. Cells* **38**, 105–111 (2015). 25537861 10.14348/molcells.2015.2301 PMC4332033

[68] Zhou, Q. T. et al. Common activation mechanism of class A GPCRs. *eLife* **8**, e50279 (2019). 10.7554/eLife.50279 PMC6954041 31855179

[69] Wess, J. Designer GPCRs as novel tools to identify metabolically important signaling pathways. *Front. Endocrinol.* **12**, 706957 (2021). 10.3389/fendo.2021.706957 PMC8329487 34354673

[70] Betke, K. M., Wells, C. A. & Hamm, H. E. GPCR mediated regulation of synaptic transmission. *Prog. Neurobiol.* **96**, 304–321 (2012). 22307060 10.1016/j.pneurobio.2012.01.009 PMC3319362

[71] Pascual-Vargas, P. & Salinas, P. C. A role for frizzled and their post-translational modifications in the mammalian central nervous system. *Front. Cell Dev. Biol.* **9**, 692888 (2021). 34414184 10.3389/fcell.2021.692888 PMC8369345

[72] Gusach, A., Maslov, I., Luginina, A., Borshchevskiy, V., Mishin, A. & Cherezov, V. Beyond structure: emerging approaches to study GPCR dynamics. *Curr. Opin. Struct. Biol.* **63**, 18–25 (2020). 32305785 10.1016/j.sbi.2020.03.004

[73] Klauer, M. J., Willette, B. K. A. & Tsvetanova, N. G. Functional diversification of cell signaling by GPCR localization. *J. Biol. Chem.* **300**, 105668 (2024). 38272232 10.1016/j.jbc.2024.105668 PMC10882132

[74] Bernhard, S. M., Han, J. & Che, T. GPCR-G protein selectivity revealed by structural pharmacology. *FEBS J.* **291**, 2784–2791 (2024). 38151714 10.1111/febs.17049 PMC11209754

[75] Zhao, L. H. et al. Structure insights into selective coupling of G protein subtypes by a class B G protein-coupled receptor. *Nat. Commun.* **13**, 6670 (2022). 36335102 10.1038/s41467-022-33851-3 PMC9637140

[76] Suomivuori, C. M. et al. Molecular mechanism of biased signaling in a prototypical G protein-coupled receptor. *Science* **367**, 881–887 (2020). 32079767 10.1126/science.aaz0326 PMC7259329

[77] Manglik, A. et al. Structure-based discovery of opioid analgesics with reduced side effects. *Nature* **537**, 185–190 (2016). 27533032 10.1038/nature19112 PMC5161585

[78] DeWire, S. M. et al. A G protein-biased ligand at the mu-opioid receptor is potently analgesic with reduced gastrointestinal and respiratory dysfunction compared with morphine. *J. Pharm. Exp. Ther.* **344**, 708–717 (2013). 10.1124/jpet.112.201616 23300227

[79] Vu, O., Bender, B. J., Pankewitz, L., Huster, D., Beck-Sickinger, A. G. & Meiler, J. The structural basis of peptide binding at class A G protein-coupled receptors. *Molecules* **27**, 210 (2021). 10.3390/molecules27010210 PMC8746363 35011444

[80] Bortolato, A., Dore, A. S., Hollenstein, K., Tehan, B. G., Mason, J. S. & Marshall, F. H. Structure of class B GPCRs: new horizons for drug discovery. *Br. J. Pharm.* **171**, 3132–3145 (2014). 10.1111/bph.12689 PMC4080969 24628305

[81] Castro, M., Nikolaev, V. O., Palm, D., Lohse, M. J. & Vilardaga, J. P. Turn-on switch in parathyroid hormone receptor by a two-step parathyroid hormone binding mechanism. *Proc. Natl Acad. Sci. USA* **102**, 16084–16089 (2005). 16236727 10.1073/pnas.0503942102 PMC1276049

[82] Hoare, S. R. Allosteric modulators of class B G-protein-coupled receptors. *Curr. Neuropharmacol.* **5**, 168–179 (2007). 19305799 10.2174/157015907781695928 PMC2656815

[83] Cary, B. P. et al. New insights into the structure and function of class B1 GPCRs. *Endocr. Rev.* **44**, 492–517 (2023). 36546772 10.1210/endrev/bnac033 PMC10166269

[84] Hilger, D. et al. Structural insights into differences in G protein activation by family A and family B GPCRs. *Science* **369**, eaba3373 (2020). 10.1126/science.aba3373 PMC7954662 32732395

[85] Knox, C. et al. DrugBank 6.0: the DrugBank Knowledgebase for 2024. *Nucleic Acids Res.* **52**, D1265–D1275 (2024). 37953279 10.1093/nar/gkad976 PMC10767804

[86] Yang, D. et al. G protein-coupled receptors: structure- and function-based drug discovery. *Signal Transduct. Target. Ther.* **6**, 7 (2021). 33414387 10.1038/s41392-020-00435-w PMC7790836

[87] Strachan, R. T. et al. Divergent transducer-specific molecular efficacies generate biased agonism at a G protein-coupled receptor (GPCR). *J. Biol. Chem.* **289**, 14211–14224 (2014). 24668815 10.1074/jbc.M114.548131 PMC4022887

[88] Wingler, L. M. et al. Angiotensin and biased analogs induce structurally distinct active conformations within a GPCR. *Science* **367**, 888–892 (2020). 10.1126/science.aay9813 PMC7171558 32079768

[89] Harris, D. M., Cohn, H. I., Pesant, S., Zhou, R. H. & Eckhart, A. D. Vascular smooth muscle G_q_ signaling is involved in high blood pressure in both induced renal and genetic vascular smooth muscle-derived models of hypertension. *Am. J. Physiol. Heart Circ. Physiol.* **293**, H3072–H3079 (2007). 17873012 10.1152/ajpheart.00880.2007

[90] Wolf, G. & Wenzel, U. O. Angiotensin II and cell cycle regulation. *Hypertension* **43**, 693–698 (2004). 14967829 10.1161/01.HYP.0000120963.09029.ca

[91] Suzuki, Y., Ruiz-Ortega, M., Lorenzo, O., Ruperez, M., Esteban, V. & Egido, J. Inflammation and angiotensin II. *Int J. Biochem Cell Biol.* **35**, 881–900 (2003). 12676174 10.1016/s1357-2725(02)00271-6

[92] Dandona, P., Dhindsa, S., Ghanim, H. & Chaudhuri, A. Angiotensin II and inflammation: the effect of angiotensin-converting enzyme inhibition and angiotensin II receptor blockade. *J. Hum. Hypertens.* **21**, 20–27 (2007). 17096009 10.1038/sj.jhh.1002101

[93] Kawai, T., Forrester, S. J., O’Brien, S., Baggett, A., Rizzo, V. & Eguchi, S. AT1 receptor signaling pathways in the cardiovascular system. *Pharm. Res.* **125**, 4–13 (2017). 10.1016/j.phrs.2017.05.008 PMC5607088 28527699

[94] Ramchandran, R. et al. Angiotensinergic stimulation of vascular endothelium in mice causes hypotension, bradycardia, and attenuated angiotensin response. *Proc. Natl Acad. Sci. USA* **103**, 19087–19092 (2006). 17148616 10.1073/pnas.0602715103 PMC1748181

[95] Zhang, H. et al. Structure of the angiotensin receptor revealed by serial femtosecond crystallography. *Cell* **161**, 833–844 (2015). 25913193 10.1016/j.cell.2015.04.011 PMC4427029

[96] Ballesteros, J. A. & Weinstein, H. Integrated methods for the construction of three-dimensional models and computational probing of structure-function relations in G protein-coupled receptors. *Methods Neurosci.* **25**, 366–428 (1995).

[97] Zhang, D. Q. et al. Structural insights into angiotensin receptor signaling modulation by balanced and biased agonists. *EMBO J.* **42**, e112940 (2023). 10.15252/embj.2022112940 PMC10233375 37038975

[98] Correa, T. D., Takala, J. & Jakob, S. M. Angiotensin II in septic shock. *Crit. Care* **19**, 98 (2015). 25886853 10.1186/s13054-015-0802-3 PMC4360936

[99] Khanna, A. et al. Angiotensin II for the treatment of vasodilatory shock. *N. Engl. J. Med.* **377**, 419–430 (2017). 28528561 10.1056/NEJMoa1704154

[100] Senatore, F. et al. FDA approval of angiotensin II for the treatment of hypotension in adults with distributive shock. *Am. J. Cardiovasc Drugs* **19**, 11–20 (2019). 30144016 10.1007/s40256-018-0297-9

[101] Brunner, H. R. The new oral angiotensin II antagonist olmesartan medoxomil: a concise overview. *J. Hum. Hypertens.* **16**, S13–S16 (2002). 10.1038/sj.jhh.1001391 11967728

[102] Molon, B. et al. T cell costimulation by chemokine receptors. *Nat. Immunol.* **6**, 465–471 (2005). 15821738 10.1038/ni1191

[103] Luster, A. D. The role of chemokines in linking innate and adaptive immunity. *Curr. Opin. Immunol.* **14**, 129–135 (2002). 11790543 10.1016/s0952-7915(01)00308-9

[104] Lederman, M. M., Penn-Nicholson, A., Cho, M. & Mosier, D. Biology of CCR5 and its role in HIV infection and treatment. *JAMA* **296**, 815–826 (2006). 16905787 10.1001/jama.296.7.815

[105] Samson, M., Labbe, O., Mollereau, C., Vassart, G. & Parmentier, M. Molecular cloning and functional expression of a new human CC-chemokine receptor gene. *Biochemistry* **35**, 3362–3367 (1996). 8639485 10.1021/bi952950g

[106] Peng, P. et al. Structure-based design of 1-heteroaryl-1,3-propanediamine derivatives as a novel series of CC-chemokine receptor 5 antagonists. *J. Med. Chem.* **61**, 9621–9636 (2018). 30234300 10.1021/acs.jmedchem.8b01077

[107] Isaikina, P. et al. Structural basis of the activation of the CC chemokine receptor 5 by a chemokine agonist. *Sci. Adv.* **7**, eabg8685 (2021). 10.1126/sciadv.abg8685 PMC8208711 34134983

[108] Wasilko, D. J. et al. Structural basis for chemokine receptor CCR6 activation by the endogenous protein ligand CCL20. *Nat. Commun.* **11**, 3031 (2020). 10.1038/s41467-020-16820-6 PMC7295996 32541785

[109] Kato, H. E. et al. Conformational transitions of a neurotensin receptor 1–G_i1_ complex. *Nature* **572**, 80–85 (2019). 31243364 10.1038/s41586-019-1337-6 PMC7065593

[110] Koehl, A. et al. Structure of the micro-opioid receptor–G_i_ protein complex. *Nature* **558**, 547–552 (2018). 29899455 10.1038/s41586-018-0219-7 PMC6317904

[111] Lieberman-Blum, S. S., Fung, H. B. & Bandres, J. C. Maraviroc: a CCR5-receptor antagonist for the treatment of HIV-1 infection. *Clin. Ther.* **30**, 1228–1250 (2008). 18691983 10.1016/s0149-2918(08)80048-3

[112] Tan, Q. et al. Structure of the CCR5 chemokine receptor–HIV entry inhibitor maraviroc complex. *Science* **341**, 1387–1390 (2013). 24030490 10.1126/science.1241475 PMC3819204

[113] Zhang, H. et al. Structural basis for chemokine recognition and receptor activation of chemokine receptor CCR5. *Nat. Commun.* **12**, 4151 (2021). 34230484 10.1038/s41467-021-24438-5 PMC8260604

[114] Baba, M. et al. A small-molecule, nonpeptide CCR5 antagonist with highly potent and selective anti-HIV-1 activity. *Proc. Natl Acad. Sci. USA* **96**, 5698–5703 (1999). 10318947 10.1073/pnas.96.10.5698 PMC21923

[115] Baba, M. et al. TAK-652 inhibits CCR5-mediated human immunodeficiency virus type 1 infection in vitro and has favorable pharmacokinetics in humans. *Antimicrob. Agents Chemother.* **49**, 4584–4591 (2005). 16251299 10.1128/AAC.49.11.4584-4591.2005 PMC1280155

[116] Stupple, P. A. et al. An imidazopiperidine series of CCR5 antagonists for the treatment of HIV: the discovery of N-(1S)-1-(3-fluorophenyl)-3-[(3-endo)-3-(5-isobutyryl-2-methyl-4,5,6,7-tetrahydro-1H-imidazo[4,5-c]pyridin-1-yl)-8-azabicyclo[3.2.1]oct-8-yl]propylacetamide (PF-232798). *J. Med. Chem.* **54**, 67–77 (2011). 10.1021/jm100978n 21128663

[117] Westby, M. & van der Ryst, E. CCR5 antagonists: host-targeted antivirals for the treatment of HIV infection. *Antivir. Chem. Chemother.* **16**, 339–354 (2005). 16329283 10.1177/095632020501600601

[118] Klibanov, O. M., Williams, S. H. & Iler, C. A. Cenicriviroc, an orally active CCR5 antagonist for the potential treatment of HIV infection. *Curr. Opin. Investig. Drugs* **11**, 940–950 (2010). 20721836

[119] Zhuang, Y. et al. Molecular recognition of morphine and fentanyl by the human mu-opioid receptor. *Cell* **185**, 4361–4375 (2022). 36368306 10.1016/j.cell.2022.09.041

[120] Manglik, A. et al. Crystal structure of the micro-opioid receptor bound to a morphinan antagonist. *Nature* **485**, 321–326 (2012). 22437502 10.1038/nature10954 PMC3523197

[121] Qu, Q. et al. Insights into distinct signaling profiles of the microOR activated by diverse agonists. *Nat. Chem. Biol.* **19**, 423–430 (2023). 36411392 10.1038/s41589-022-01208-y PMC11098091

[122] Juppner, H. et al. A G protein-linked receptor for parathyroid hormone and parathyroid hormone-related peptide. *Science* **254**, 1024–1026 (1991). 1658941 10.1126/science.1658941

[123] Fan, Y. et al. Parathyroid hormone 1 receptor is essential to induce FGF23 production and maintain systemic mineral ion homeostasis. *FASEB J.* **30**, 428–440 (2016). 26428657 10.1096/fj.15-278184 PMC4684518

[124] Kir, S. et al. Tumour-derived PTH-related protein triggers adipose tissue browning and cancer cachexia. *Nature* **513**, 100–104 (2014). 25043053 10.1038/nature13528 PMC4224962

[125] Neer, R. M. et al. Effect of parathyroid hormone (1–34) on fractures and bone mineral density in postmenopausal women with osteoporosis. *N. Engl. J. Med* **344**, 1434–1441 (2001). 11346808 10.1056/NEJM200105103441904

[126] Kobayashi, K. et al. Endogenous ligand recognition and structural transition of a human PTH receptor. *Mol. Cell* **82**, 3468–3483 (2022). 35932760 10.1016/j.molcel.2022.07.003

[127] Ehrenmann, J. et al. High-resolution crystal structure of parathyroid hormone 1 receptor in complex with a peptide agonist. *Nat. Struct. Mol. Biol.* **25**, 1086–1092 (2018). 30455434 10.1038/s41594-018-0151-4

[128] Cary, B. P. et al. Molecular insights into peptide agonist engagement with the PTH receptor. *Structure* **31**, 668–676 (2023). 37148874 10.1016/j.str.2023.04.002

[129] Frolik, C. A. et al. Anabolic and catabolic bone effects of human parathyroid hormone (1–34) are predicted by duration of hormone exposure. *Bone* **33**, 372–379 (2003). 13678779 10.1016/s8756-3282(03)00202-3

[130] Tamura, T. et al. Identification of an orally active small-molecule PTHR1 agonist for the treatment of hypoparathyroidism. *Nat. Commun.* **7**, 13384 (2016). 27857062 10.1038/ncomms13384 PMC5120204

[131] Kobayashi, K. et al. Class B1 GPCR activation by an intracellular agonist. *Nature* **618**, 1085–1093 (2023). 37286611 10.1038/s41586-023-06169-3 PMC10307627

[132] Zhao, L. H. et al. Conserved class B GPCR activation by a biased intracellular agonist. *Nature* **621**, 635–641 (2023). 37524305 10.1038/s41586-023-06467-w

[133] Drucker, D. J. Mechanisms of action and therapeutic application of glucagon-like peptide-1. *Cell Metab.* **27**, 740–756 (2018). 29617641 10.1016/j.cmet.2018.03.001

[134] Drucker, D. J., Habener, J. F. & Holst, J. J. Discovery, characterization, and clinical development of the glucagon-like peptides. *J. Clin. Invest.* **127**, 4217–4227 (2017). 29202475 10.1172/JCI97233 PMC5707151

[135] Drucker, D. J., Philippe, J., Mojsov, S., Chick, W. L. & Habener, J. F. Glucagon-like peptide I stimulates insulin gene expression and increases cyclic AMP levels in a rat islet cell line. *Proc. Natl Acad. Sci. USA* **84**, 3434–3438 (1987). 3033647 10.1073/pnas.84.10.3434 PMC304885

[136] Hvidberg, A., Nielsen, M. T., Hilsted, J., Orskov, C. & Holst, J. J. Effect of glucagon-like peptide-1 (proglucagon 78–107amide) on hepatic glucose production in healthy man. *Metabolism* **43**, 104–108 (1994). 8289665 10.1016/0026-0495(94)90164-3

[137] Turton, M. D. et al. A role for glucagon-like peptide-1 in the central regulation of feeding. *Nature* **379**, 69–72 (1996). 8538742 10.1038/379069a0

[138] Flint, A., Raben, A., Astrup, A. & Holst, J. J. Glucagon-like peptide 1 promotes satiety and suppresses energy intake in humans. *J. Clin. Invest.* **101**, 515–520 (1998). 9449682 10.1172/JCI990 PMC508592

[139] Wu, F. et al. Full-length human GLP-1 receptor structure without orthosteric ligands. *Nat. Commun.* **11**, 1272 (2020). 32152292 10.1038/s41467-020-14934-5 PMC7062719

[140] Underwood, C. R. et al. Crystal structure of glucagon-like peptide-1 in complex with the extracellular domain of the glucagon-like peptide-1 receptor. *J. Biol. Chem.* **285**, 723–730 (2010). 19861722 10.1074/jbc.M109.033829 PMC2804221

[141] Liang, Y. L. et al. Phase-plate cryo-EM structure of a biased agonist-bound human GLP-1 receptor–G_s_ complex. *Nature* **555**, 121–125 (2018). 29466332 10.1038/nature25773

[142] Li, Y. et al. Structural analysis of the dual agonism at GLP-1R and GCGR. *Proc. Natl Acad. Sci. USA* **120**, e2303696120 (2023). 37549266 10.1073/pnas.2303696120 PMC10438375

[143] Zhao, F. et al. Structural insights into multiplexed pharmacological actions of tirzepatide and peptide 20 at the GIP, GLP-1 or glucagon receptors. *Nat. Commun.* **13**, 1057 (2022). 35217653 10.1038/s41467-022-28683-0 PMC8881610

[144] Ma, H. et al. Structural insights into the activation of GLP-1R by a small molecule agonist. *Cell Res* **30**, 1140–1142 (2020). 32724086 10.1038/s41422-020-0384-8 PMC7784854

[145] Zhang, Y. et al. Cryo-EM structure of the activated GLP-1 receptor in complex with a G protein. *Nature* **546**, 248–253 (2017). 28538729 10.1038/nature22394 PMC5587415

[146] Cong, Z. et al. Molecular features of the ligand-free GLP-1R, GCGR and GIPR in complex with G_s_ proteins. *Cell Discov.* **10**, 18 (2024). 38346960 10.1038/s41421-024-00649-0 PMC10861504

[147] Eng, J., Kleinman, W. A., Singh, L., Singh, G. & Raufman, J. P. Isolation and characterization of exendin-4, an exendin-3 analogue, from Heloderma suspectum venom. Further evidence for an exendin receptor on dispersed acini from guinea pig pancreas. *J. Biol. Chem.* **267**, 7402–7405 (1992). 1313797

[148] Deganutti, G. et al. Dynamics of GLP-1R peptide agonist engagement are correlated with kinetics of G protein activation. *Nat. Commun.* **13**, 92 (2022). 35013280 10.1038/s41467-021-27760-0 PMC8748714

[149] Agerso, H., Jensen, L. B., Elbrond, B., Rolan, P. & Zdravkovic, M. The pharmacokinetics, pharmacodynamics, safety and tolerability of NN2211, a new long-acting GLP-1 derivative, in healthy men. *Diabetologia* **45**, 195–202 (2002). 11935150 10.1007/s00125-001-0719-z

[150] Madsen, K. et al. Structure–activity and protraction relationship of long-acting glucagon-like peptide-1 derivatives: importance of fatty acid length, polarity, and bulkiness. *J. Med. Chem.* **50**, 6126–6132 (2007). 17975905 10.1021/jm070861j

[151] Lau, J. et al. Discovery of the once-weekly glucagon-like peptide-1 (GLP-1) analogue semaglutide. *J. Med. Chem.* **58**, 7370–7380 (2015). 26308095 10.1021/acs.jmedchem.5b00726

[152] Knudsen, L. B. & Lau, J. The discovery and development of liraglutide and semaglutide. *Front. Endocrinol.* **10**, 155 (2019). 10.3389/fendo.2019.00155 PMC6474072 31031702

[153] Zhang, X., Belousoff, M. J., Liang, Y. L., Danev, R., Sexton, P. M. & Wootten, D. Structure and dynamics of semaglutide- and taspoglutide-bound GLP-1R-Gs complexes. *Cell Rep.* **36**, 109374 (2021). 34260945 10.1016/j.celrep.2021.109374

[154] Coskun, T. et al. LY3298176, a novel dual GIP and GLP-1 receptor agonist for the treatment of type 2 diabetes mellitus: from discovery to clinical proof of concept. *Mol. Metab.* **18**, 3–14 (2018). 30473097 10.1016/j.molmet.2018.09.009 PMC6308032

[155] Knerr, P. J., Mowery, S. A., Finan, B., Perez-Tilve, D., Tschop, M. H. & DiMarchi, R. D. Selection and progression of unimolecular agonists at the GIP, GLP-1, and glucagon receptors as drug candidates. *Peptides* **125**, 170225 (2020). 31786282 10.1016/j.peptides.2019.170225

[156] Li, W. et al. Structural insights into the triple agonism at GLP-1R, GIPR and GCGR manifested by retatrutide. *Cell Discov.* **10**, 77 (2024). 39019866 10.1038/s41421-024-00700-0 PMC11255275

[157] Campbell, J. E., Muller, T. D., Finan, B., DiMarchi, R. D., Tschop, M. H. & D’Alessio, D. A. GIPR/GLP-1R dual agonist therapies for diabetes and weight loss-chemistry, physiology, and clinical applications. *Cell Metab.* **35**, 1519–1529 (2023). 37591245 10.1016/j.cmet.2023.07.010 PMC10528201

[158] Yin, J. et al. Structure and ligand-binding mechanism of the human OX1 and OX2 orexin receptors. *Nat. Struct. Mol. Biol.* **23**, 293–299 (2016). 26950369 10.1038/nsmb.3183

[159] Yin, J., Mobarec, J. C., Kolb, P. & Rosenbaum, D. M. Crystal structure of the human OX2 orexin receptor bound to the insomnia drug suvorexant. *Nature* **519**, 247–250 (2015). 25533960 10.1038/nature14035

[160] Hellmann, J. et al. Structure-based development of a subtype-selective orexin 1 receptor antagonist. *Proc. Natl Acad. Sci. USA* **117**, 18059–18067 (2020). 32669442 10.1073/pnas.2002704117 PMC7395530

[161] Harding, S. D. et al. The IUPHAR/BPS Guide to PHARMACOLOGY in 2024. *Nucleic Acids Res.* **52**, D1438–D1449 (2024). 37897341 10.1093/nar/gkad944 PMC10767925

[162] Garces, F. et al. Molecular insight into recognition of the CGRPR complex by migraine prevention therapy aimovig (erenumab). *Cell Rep.* **30**, 1714–1723 (2020). 32049005 10.1016/j.celrep.2020.01.029

[163] Shi, L. et al. Pharmacologic characterization of AMG 334, a potent and selective human monoclonal antibody against the calcitonin gene-related peptide receptor. *J. Pharm. Exp. Ther.* **356**, 223–231 (2016). 10.1124/jpet.115.227793 26559125

[164] Poduslo, J. F., Curran, G. L. & Berg, C. T. Macromolecular permeability across the blood–nerve and blood–brain barriers. *Proc. Natl Acad. Sci. USA* **91**, 5705–5709 (1994). 8202551 10.1073/pnas.91.12.5705 PMC44065

[165] Pellitteri, G. et al. Erenumab impact on sleep assessed with questionnaires and home-polysomnography in patients with migraine: the ERESON study. *Front. Neurol.* **13**, 869677 (2022). 35645951 10.3389/fneur.2022.869677 PMC9136084

[166] Ohlsson, L., Haanes, K. A., Kronvall, E., Xu, C., Snellman, J. & Edvinsson, L. Erenumab (AMG 334), a monoclonal antagonist antibody against the canonical CGRP receptor, does not impair vasodilatory or contractile responses to other vasoactive agents in human isolated cranial arteries. *Cephalalgia* **39**, 1745–1752 (2019). 31366221 10.1177/0333102419867282

[167] Chari, A. et al. Talquetamab, a T-cell-redirecting GPRC5D bispecific antibody for multiple myeloma. *N. Engl. J. Med*. **387**, 2232–2244 (2022). 36507686 10.1056/NEJMoa2204591

[168] Rasmussen, S. G. et al. Structure of a nanobody-stabilized active state of the β_2_ adrenoceptor. *Nature* **469**, 175–180 (2011). 21228869 10.1038/nature09648 PMC3058308

[169] Abramson, J. et al. Accurate structure prediction of biomolecular interactions with AlphaFold 3. *Nature* **630**, 493–500 (2024). 38718835 10.1038/s41586-024-07487-w PMC11168924

[170] He, X. H., Li, J. R., Shen, S. Y. & Xu, H. E. AlphaFold3 versus experimental structures: assessment of the accuracy in ligand-bound G protein-coupled receptors. *Acta Pharmacol. Sin.* **46**, 1111–1122 (2024). 10.1038/s41401-024-01429-y PMC11950431 39643640

[171] Krishna, R. et al. Generalized biomolecular modeling and design with RoseTTAFold All-Atom. *Science***384**, eadl2528 (2024).38452047 10.1126/science.adl2528

[172] Balyschew, N., Yushkevich, A., Mikirtumov, V., Sanchez, R. M., Sprink, T. & Kudryashev, M. Streamlined structure determination by cryo-electron tomography and subtomogram averaging using TomoBEAR. *Nat. Commun.* **14**, 6543 (2023). 37848413 10.1038/s41467-023-42085-w PMC10582028

[173] Liu, A., Liu, Y. & Ye, R. D. Structural basis of CXCR4 assembly and regulation. *Cell Rep.* **44**, 115255 (2025). 39891908 10.1016/j.celrep.2025.115255

